# Hypoxia‐Induced Metabolic and Functional Changes in Oral CSCs: Implications for Stemness and Viability Modulation Through BNIP3‐Driven Mitophagy

**DOI:** 10.1111/jcmm.70400

**Published:** 2025-02-13

**Authors:** Xin Li, Hitesh Singh Chaouhan, Shao‐Hua Yu, I‐Kuan Wang, Tung‐Min Yu, Ya‐Wen Chuang, Kuen‐Bao Chen, Feng‐Yen Lin, Michael Yuan‐Chien Chen, Che‐Hao Hsu, Kuo‐Ting Sun, Chi‐Yuan Li

**Affiliations:** ^1^ Graduate Institute of Biomedical Sciences China Medical University Taichung Taiwan; ^2^ Department of Emergency Medicine China Medical University Hospital Taichung Taiwan; ^3^ Division of Nephrology China Medical University Hospital Taichung Taiwan; ^4^ Department of Internal Medicine, School of Medicine China Medical University Taichung Taiwan; ^5^ Division of Nephrology, Department of Internal Medicine Taichung Veterans General Hospital Taichung Taiwan; ^6^ School of Medicine China Medical University Taichung Taiwan; ^7^ Department of Post‐Baccalaureate Medicine, College of Medicine National Chung Hsing University Taichung Taiwan; ^8^ Department of Anesthesiology China Medical University Hospital Taichung Taiwan; ^9^ Taipei Heart Institute Taipei Medical University Taipei Taiwan; ^10^ Division of Cardiology and Cardiovascular Research Center Taipei Medical University Hospital Taipei Taiwan; ^11^ Department of Internal Medicine, College of Medicine, School of Medicine Taipei Medical University Taipei Taiwan; ^12^ School of Dentistry China Medical University Taichung Taiwan; ^13^ Department of Dentistry China Medical University Hospital Taichung Taiwan; ^14^ Department of Anesthesiology Tungs' Taichung Metroharbor Hospital Taichung Taiwan; ^15^ Department of Pediatric Dentistry China Medical University Hospital Taichung Taiwan

**Keywords:** autophagy, BNIP3/‐L, cancer stem cells, Mitophagy, oral squamous cell carcinoma, oxidative phosphorylation

## Abstract

Oral squamous cell carcinomas (OSCCs), like several solid tumours, contain heterogeneous subpopulations of a small subset of cancer cells, termed cancer stem cells (CSCs), that are highly relevant to cancer metastasis and invasive properties. CSCs have also shown a high capacity to survive against various stressful environments, such as hypoxia. However, the molecular underpinnings behind the high potential of CSCs to survive under this stress remain unclear. The current study aimed to investigate the significance of autophagy systems in oral CSC maintenance and survival under stress conditions. Human OSCC cell lines OECM‐1 and OECM‐1 CSCs were cultured in different hypoxic time periods for proliferation and cytotoxicity analyses. The stemness property of CSCs is evaluated by sphere formation, transwell and wound healing assays protein expression of stemness, and epithelial‐to‐mesenchymal transition markers. Mitochondrial functions, including mitochondrial ROS generation, mitochondria dynamics, mitophagy, and mitochondrial metabolism (glycolysis and oxidative phosphorylation [OXPHOS]) were examined by western blotting, immunohistochemistry, and XF‐seahorse assays, respectively. Under hypoxia, oral CSCs showed a higher proliferation rate with increased invasion/migration/EMT properties than OECM‐1 cells. Further, hypoxia‐induced BNIP3‐driven mitophagy was activated in OECM‐1 CSCs than in OECM‐1 cells, which also triggered a metabolic shift towards OXPHOS, and BNIP3/‐L silencing by siRNA significantly attenuated OECM‐1 CSCs stemness features. TCGA data analyses also revealed a higher BNIP3 expression in head and neck squamous carcinoma patients' tumour samples associated with lower patient survival. Collectively, our results revealed a BNIP3/‐L‐driven autophagy contributes to the OECM‐1 CSCs stemness features under hypoxia, suggesting a novel therapeutic strategy involving BNIP3 and autophagy inhibition in oral CSCs.

## 1 Introduction

Oral cancer, the most common type of head and neck malignancy, is the eighth most prevalent cancer worldwide and accounts for 10%–12% of cancer in developing nations [1]. Oral squamous cell carcinomas (OSCCs) account for more than 90% of oral cancer and originate from any part of the oral tissues, including the tongue, lips, and gingivobuccal complex area. According to a GLOBOCAN 2020 report, an estimated 657,000 new cases and 330,000 deaths annually are due to the prevalence of oral cancer in 2020, and these new cases are expected to double globally by 2035, according to the WHO reports [1, 2, 3]. The risk factors for OSCC incidence include chronic inflammation and irritation from betel nut chewing, tobacco smoking, alcohol drinking, and viral infection [4, 5]. Earlier studies have reported that even after the use of the advances in the diagnosis and treatment of OSCCs, the prognosis of OSCCs remains poor, with a 5‐year survival rate primarily because of the higher invasion, metastasis, and recurrence rates or complications within 5 years under current therapies [4, 6]. Therefore, a better understanding of cellular and molecular mechanisms that promote OSCC tumorigenesis or cancer growth is needed to explore novel therapeutic molecular targets to improve treatments and prognosis of OSCC patients.

Recent studies have reported that each tumour contains a unique subpopulation of a small subset of functionally heterogeneous cells that undergo not only proliferation but also differentiation and maturation to a certain degree, termed cancer stem cells (CSCs) [7]. Further, studies reported CSC exhibited a high self‐renewal property that can differentiate into the daughter cancer cells, are less sensitive to radiotherapy and chemotherapeutic agents, and are relevant to the cancer re‐initiating, metastasis, and invasiveness properties [8, 9, 10]. The heterogeneity of OSCCs has also been studied; however, the existence of CSCs from OSCCs has not been well characterised. In addition, CSCs have also shown a high capacity to survive against various stressful environments, such as hypoxia and starvation, resulting in an increase in the proportion of CSCs [11, 12]. However, the molecular mechanisms related to the high potential of CSCs to survive under these stress conditions have not yet been cleared.

Hypoxia‐induced alteration of mitochondrial functions and mitochondrial metabolism has recently become a hot topic in cancer research. Mitochondria‐specific autophagy (hereafter termed mitophagy), one of the critical components of mitochondria quality control, selectively removes the impaired mitochondria and protein aggregates to autophagosomes in a lysosome‐dependent manner, thus maintaining mitochondrial homeostasis, cell survival, and limiting oxidative stress [13, 14]. In recent years, the molecular functions of mitophagy pathways have been extensively studied. There are two major types of mitophagy regulatory pathways; one is the PTEN‐induced putative kinase 1 (PINK1)‐Parkin non‐receptor‐mediated pathway, and the other is the Bcl2 interacting protein 3 (BNIP3), NIP3‐like protein X (NIX), and FUN14 domain‐containing protein 1 (FUNDC1) receptor‐mediated pathway. In the non‐receptor‐dependent mitophagy pathway, the loss of mitochondrial membrane potential (MMP, ψΔm) leads to PINK1 protein stabilisation at the outer mitochondrial membrane (OMM) and subsequently recruits Parkin to assist in the removal of impaired mitochondria. In the receptor‐mediated pathway, BNIP3, NIX, and FUNDC1 are OMM‐localised as mitophagy receptors in response to hypoxia and starvation stimuli, which further promote the attachment of autophagosomes to the OMM through their light chain‐3 (LC3) interacting region motif at the phagophore membrane. Inefficient induction of mitophagy due to impairment of either pathway has been shown to leave dysfunctional mitochondria and render cancer cells more sensitive to stressful conditions such as hypoxia, oxidative stress, and chemotherapeutic cytotoxicity [15, 16]. Several studies have reported that mitophagy plays an important role in the development and maintenance of stemness in CSCs in various types of cancer, including pancreatic ductal adenocarcinoma [17, 18], colon cancer [19, 20], breast cancer [21, 22], lung cancer [23], and hepatocarcinoma [24]. Many studies have correlated the upregulation of BNIP3/‐L with aggressive malignant behaviour in various types of cancer, such as prostate [25], colorectal [26], breast [27], and endometrial [28]. Some studies also reported that inhibition of BNIP3‐dependent mitophagy curtailed chemotherapy resistance in osteosarcoma and ovarian cancer [29], as well as colorectal CSCs [20]. Furthermore, Dykstra et al. [30] reported that upon exposure to hypoxia, activation of receptor‐driven mitophagy clears impaired mitochondria and maintains mitochondrial homeostasis and cell survival in human leukaemia stem cells. However, whether and how BNIP3/‐L‐driven mitophagy promotes the progression and survival of oral carcinoma CSCs under hypoxia remains to be elucidated. Thus, more detailed experimental molecular mechanisms underlying the functions of mitophagy in the acquisition and maintenance of cancer stemness in hypoxia‐exposed OSCCs are worthy of further study.

In the present study, mitophagy is one of the main pathways activated in hypoxia‐treated oral CSCs. Importantly, we investigated that higher levels of BNIP3/‐L in hypoxic oral CSCs, mainly through phosphorylation at Ser616 of DRP1, are crucial factors responsible for increased proliferation and stemness features of oral CSCs. Inhibition of mitophagy arising from silencing BNIP3 expression led to higher levels of mitochondrial ROS, reduced cancer cell survival, and ultimately inhibited tumour progression and metastasis. We also hereby define a novel molecular mechanism by which BNIP3‐driven autophagy sustains oral CSC progression and metastasis under hypoxic conditions through a mixed glycolytic/OXPHOS metabolism with a prevalent increase in OXPHOS levels, pushing them to become more reliant on mitochondrial oxidative metabolism for survival, and further targeting of this pathway could restrict the survival of CSCs, resulting in the development of new therapeutic strategies for cancer cells.

## 2 Materials and Methods

### 2.1 Cell Culture and Hypoxia Treatment

The human oral squamous carcinoma OECM‐1 cell line was purchased from the American Type Culture Collection (ATCC, Manassas, VA, USA) and maintained in our laboratory for the current study. For conventional cell culture, 1 × 10^5^ cells were initially seeded onto 60 mm culture plates to a confluence of approximately 80%–90% and maintained in Dulbecco's modified Eagle's medium‐low glucose (DMEM; Merck Millipore, Germany) containing charcoal‐treated 10% FBS (Gibco Life Technologies, USA), 100 U/mL penicillin/streptomycin, nonessential amino acid, sodium bicarbonate, and 2 mM l‐glutamine. Additionally, oral cancer stem cells (OECM‐1 CSCs) were derived from the human OECM‐1 cell lines by culturing OECM‐1 cells in serum‐free medium (SFM) containing bFGF and EGF. After being cultured for approximately 2 weeks, a subset of OECM‐1 cells gradually began to form spherical cell aggregates. Furthermore, the cell population was trypsinised to separate and collect spheres from non‐spheres, and subsequently both subpopulations were washed three times with DPBS to quench the trypsin reaction. Under normoxic conditions, cells were cultured in an 80%–90% humidified incubator (Thermo Fisher Scientific, USA) containing 5% CO_2_ and 95% air (21% O_2_) at 37°C. While for hypoxia exposure, seeded cells were placed in a modulator incubation system (Oxygen Sensors, Gladwyne, PA) containing 1% O_2_ (hypoxic condition) with 5% CO_2_ and 94% N_2_ at 37°C, as followed by previously published methods [31].

### 2.2 Cell Proliferation Assay

To evaluate the hypoxia effects on the proliferation of OECM‐1 and OECM‐1 CSCs, a cell counting kit‐8 (CCK‐8; BD Biosciences, USA) assay was used to determine the cell proliferation efficacy according to the manufacturer's instructions. Briefly, both OECM‐1 and OECM‐1 CSCs cells (with a final density of 1 **×** 10^4^ cells/mL) were seeded in triplicates in DMEM low glucose with 10% FBS in a 96‐well plate under either normoxic conditions (21% O_2_) or hypoxic conditions (1% O_2_) for variable times (0, 6, 16 and 24 h). After hypoxia exposure, the media was removed, followed by adding 10.0 μL CCK‐8 reagents with free serum medium to each 96‐well plates then placed for 4–5 h at 37°C in the incubator. At last, the absorbance rate in each well was recorded at 450 nm with the 96‐well plate's microplate reader, and the relative cell viability was calculated using untreated cells as a control (rate expressed as fold change).

### 2.3 Lactate Dehydrogenase (LDH) Assay

The cytotoxicity induced by the hypoxia treatment was assessed by detecting the release of LDH from OECM‐1 and OECM‐1 CSCs using the LDH Cytotoxicity Assay Kit (Takara Bio Inc., Japan) according to the manufacturer's protocol. In brief, the cells from both cell lines were seeded at a final density of 1 **×** 10^4^ cells/mL in triplicates in DMEM low glucose with 10% FBS in a 96‐well plate under either normoxic conditions (21% O_2_) or hypoxic conditions (1% O_2_) for varying periods (0, 6, 16, and 24 h). After hypoxic exposure, 100.0 μL of the supernatant was transferred from each well into corresponding 96‐well plates and washed three times with 1 × PBS. After that, 0.1% Triton‐X‐100 in PBS was added to the supernatant in each well to completely lyse the cell membranes and was kept for 10 min at 37°C. Next, 100.0 μL of the lysed supernatant was collected and incubated with a substrate reaction mixture (reagents A and B; 1:5) from the LDH kit. After incubation at 37°C for 15 min, the resultant solution mixture was mixed with reagent C and held at the same temperature for 15 min. Further, the reaction was stopped by adding termination solutions, 1 N HCL, and absorbance was recorded at 450 nm using the ELISA‐based multiwell plate reader. The relative cell cytotoxicity was calculated using untreated cells as a control (rate expressed as fold change).

### 2.4 Sphere‐Formation Assay

Sphere formation assay was carried out based on the earlier published procedure [32]. In brief, OECM‐1 and OECM‐1 CSC cells (1 × 10^5^ cells/plate) were seeded in 60 mm culture plates under 24 h hypoxia exposure, then detached from the culture plates and harvested. Further, cells were digested for 30 min at 37°C to create a single‐cell suspension, after which the single cells (5000 cells/well) were re‐seeded and cultivated in serum‐free DMEM medium supplemented with 10 ng/mL bFGF, 20 ng/mL EGF, and 1 × Insulin‐Transferrin‐Selenium‐A (Gibco, USA) in ultralow attachment six‐well plates (Lifesciences, USA) under normoxic conditions. After 10 days, the spheres were analysed under the Nikon‐TH100 inverse microscope and were counted as primary and secondary spheres (≥ 30 cells in the spheres are considered full spheres).

### 2.5 Cell Migration and Invasion Assays

For the migration assay, the OECM‐1 and OECM‐1 CSC cells (3 × 10^5^ cells/plate) were initially seeded onto 60‐mm culture plates to a confluence of ~80%. An artificial wound was created by vertically scratching with the end of a 200 μL pipette tip. Detached cells were then removed by washing with 1 mL of 1 × PBS and the cells were incubated with fresh DMEM‐low glucose serum‐free medium at 37°C in an incubator under normoxic or hypoxic conditions for variable times (0, 6, and 24 h). Cell migration was monitored and visualised under a phase‐contrast microscope at 10× magnification at 0, 6, and 24 h post‐exposure to hypoxia and was quantified using Image J software (version 1.53t, NIH, Bethesda, USA).

The invasion assay was carried out in 24‐transwell chambers (Millipore Sigma, Germany) according to an earlier published procedure [32]. Briefly, 1 × 10^5^ cells of OECM‐1 and OECM‐1 CSC were cultured and re‐suspended in 200 μL of DMEM low glucose serum‐free media before being seeded on the Matrigel top chamber. The lower chamber of the 24‐transwell was filled with 500 μL of 10% FBS‐supplemented DMEM media. The cell suspension was applied onto the Matrigel membrane and then incubated for 24 h under hypoxia or normoxia conditions at 37°C. Cells migrated through the Matrigel chamber, and the filter was fixed with 4% paraformaldehyde for 30 min, followed by washing 3 times in 1 × PBS solution. Finally, cells were stained with 0.2% crystal violet solution for 30 min, and images were captured using a microscope (Nikon).

### 2.6 Assessment of Intracellular ROS Level

The general level of ROS was examined in the OECM‐1 and OECM‐1 CSCs cells using the reagent 2′,7′‐dichlorodihydrofluorescein diacetate (H_2_‐DCFDA; Invitrogen, USA), which was later oxidised into highly fluorescent 2′,7′‐dichlorofluorescein (DCF) through a reaction with ROS. After hypoxia exposure (0, 6, and 24 h), the cells were washed with 1 × PBS and then incubated with 10.0 μM of DCFH‐DA diluted in serum‐free media in the dark at 37°C for 30 min. After incubation, the DCFH‐DA dye was removed, followed by washing the cells three times with serum‐free media and resuspending them in 1 × PBS solution. Finally, DCF fluorescence images were analysed using a confocal laser‐scanning microscope (Leica TCS‐SPE, Nussloch, Germany). The relative intensity of DCF fluorescence staining was quantified using Image J software (version 1.53t, NIH, Bethesda, USA).

### 2.7 Measurement of Intracellular Mitochondrial Superoxide ($O_{2}^{∙-}$) Radical Level

MitoSOX fluorescent dye was used to measure in vivo mitochondrial $O_{2}^{∙-}$ radical formation in OECM‐1 and OECM‐1 CSC cells, which is a live cell‐permeable reagent that specifically targets mitochondria and shows red fluorescence after being oxidised through the generation of mitochondrial $O_{2}^{∙-}$ radicals. Briefly, 1 × 10^5^ cells from both cell lines were seeded in each well of 8‐chamber slides. After varying periods of hypoxia exposure, the cells were washed with 1 × PBS and then incubated with 5.0 μM MitoSOX Red dye for 15 min in a 37°C incubator with 5% CO_2_. After washing with 1 × PBS three times for 5 min, the cells were mounted in DAPI fluorescence mounting media and immediately imaged using a confocal laser scanning fluorescence microscope (Leica TCS‐SPE Microsystems, Wetzlar, Germany) with a 63× objective.

### 2.8 Western Blot Assay

To examine protein expression, the method of Chaouhan et al. [33] was adopted with minor variations. In brief, total protein from the OECM‐1 and OECM‐1 CSCs cells was extracted using ice‐cold RIPA cell lysis and extraction buffer containing 50 mM Tris–HCl (pH 7.4), 150 mM NaCl, 1.0 mM EDTA, 1% NP40, 0.1% SDS, and 1 mM β‐mercaptoethanol supplemented with a cocktail of proteinase‐K inhibitor and phosphatase inhibitor. It was then placed on ice for 30 min to ensure complete cell lysis. After centrifugation at 14,000 *g* for 20 min at 4°C, the total protein fraction was transferred to a new Eppendorf tube, and protein concentration was determined using a Bradford protein assay kit (Bio‐Rad, USA) following the manufacturer's protocol. Next, an equal amount of protein was heated at 95°C for 5 min in an appropriate proportion of 5× sample buffer (10% SDS, 50% glycerol, 0.1% bromophenol blue, 250 mM Tris–HCl [pH 6.8], and 5% β‐mercaptoethanol) and separated using 10%–15% SDS‐PAGE at a constant 80 V for an appropriate time. After electrophoresis, proteins were transferred to a PVDF membrane (Millipore Corporation Ltd.) in a wet‐dry state using a Trans‐Blot Bio‐Rad wet‐dry transfer system (Bio‐Rad Laboratories, USA), followed by blocking with 5% w/v non‐fat dry skim milk in 1 × TBS‐T (1 × transfer buffer saline‐0.1% Tween‐20) for 2 h at room temperature. The membrane was probed with indicated primary antibodies at the appropriate dilution (see detailed information in Table 1) overnight at 4°C, followed by incubation with HRP‐linked secondary antibodies for 2 h at room temperature. The specific proteins on the membrane were detected using an enhanced chemiluminescent signal (ECL) substrate kit (GE Healthcare, USA) and visualised as bands on an Image Quant LAS4000 Imager system (GE Healthcare). Finally, the densitometry analysis of bands was carried out with the Image J software (version 1.53t, NIH, Bethesda, USA), with β‐ACTIN (1:1000) used as a loading control and an endogenous control.

**TABLE 1:** Abbreviated list of primary antibodies and their dilutions used for western blotting and immunocytochemistry in the present study.

Antibodies	Observed molecular weight (MW)	Dilution	Host	Company	Catalogue no. (#)
Stemness markers					
CD44	82 kDa	1:1000	Rabbit	ABclonal	A12410
SOX2	34 kDa	1:1000	Rabbit	Genetex	GTX101507
NANOG	40 kDa	1:1000	Rabbit	Genetex	GTX100863
OCT4	39 kDa	1:1000	Rabbit	Genetex	GTX100468
EMT markers					
FIBRONECTIN	263 kDa	1:1000	Rabbit	Genetex	GTX112794
E‐CADHERIN	97 kDa	1:1000	Rabbit	Genetex	GTX61329
GSK3‐α/β	51/46 kDa	1:1000	Rabbit	R&D Systems	Af2157
VIMENTIN	54 kDa	1:1000	Rabbit	Genetex	GTX100619
SNAIL	29 kDa	1:500	Rabbit	ABclonal	A5243
SLUG	30 kDa	1:1000	Rabbit	ABclonal	A1057
TWIST	21 kDa	1:1000	Rabbit	Genetex	GTX127310
Mitochondrial dynamics (fusion and fission) markers					
MFN1	80 kDa	1:1000	Rabbit	Cell Signaling	14739
MFN2	78 kDa	1:1000	Rabbit	Cell Signaling	9482
OPA	80/90 kDa	1:1000	Rabbit	Cell Signaling	80471
pDRP1	78/86 kDa	1:1000	Rabbit	Cell Signaling	8570S
DRP1	78/86 kDa	1:1000	Rabbit	Cell Signaling	3455S
Mitophagy markers					
BNIP3	24/30 kDa	1:500	Rabbit	Genetex	GTX64429
BNIP3L	19 kDa	1:500	Rabbit	Genetex	GTX111876
LC3	17–21 kDa	1:500	Rabbit	Novus	Nb100‐2220
p‐PARKIN	52 kDa	1:1000	Rabbit	BiOrbyt	orb312554
PARKIN	52 kDa	1:1000	Mouse	Cell Signaling	4211S
PINK1	50/60 kDa	1:1000	Rabbit	Cell Signaling	6946S
β‐Actin	42 kDa	1:1000	Mouse	Sigma Aldrich	Si5441a
Immunocytochemistry					
BNIP3	—	1:200	Rabbit	Genetex	GTX64429
MFN1	—	1:200	Rabbit	Genetex	GTX111876
LC3	—	1:200	Rabbit	Novus	Nb100‐2220
p‐DRP1 (S616)	—	1:200	Rabbit	Cell Signaling	3455S
Ki67	—	1:200	Rabbit	ABclonal	A2094
Cell death marker					
Cleaved caspase‐3 (p17)	—	1:100	Rabbit	Cell Signaling	9661

### 2.9 Immunofluorescence (IF) Assay and Analysis

For immunofluorescence analysis, cells of both OECM‐1 and OECM‐1 CSCs (with a final density of 5 **×** 10^3^ cells/mL) were plated on a coverslip in a 6‐well plate and cultured in a standard medium with 10% FBS either under normoxic conditions (21% O_2_) or hypoxic conditions (1% O_2_) for variable periods (0, 6, 16, and 24 h) in an incubator at 37°C. After the hypoxia exposure, the medium was removed, followed by washing the cells three times with 1 × PBS for 5 min at room temperature. Next, cells were fixed with 4% PFA in 1 × PBS for 30 min at 24°C ± 1°C and then permeabilized using 0.1% Triton‐X‐100 in 1 × PBS (1 × PBS‐T) solution at room temperature for 15 min. After blocking of unspecific binding sites in 4% bovine serum albumin in 1 × PBS‐T solution for 2 h at 25°C ± 2°C, cells were incubated with the indicated primary antibodies at an appropriate dilution (see detailed information in Table 1) overnight at 4°C. The next day, the cells were washed three times with 1 × PBS and then stained with Alexa Fluor‐488 and ‐546 conjugated goat anti‐rabbit and ‐mouse (1:400; Invitrogen, USA) secondary antibodies for 2 h at room temperature. Finally, the slides were mounted with fluorescence mounting media (Thermo Scientific, USA) for 5 min at 24°C ± 1°C, and images were captured using a confocal laser scanning microscope (Leica TCS‐SPE Microsystems, Germany) with a 63× objective. For LC3B puncta quantification in both OECM‐1 and OECM‐1 CSCs, Thirty cells were manually analysed for each sample from three independent biological replicates. The numbers of puncta were counted by setting the corresponding secondary antibody fluorescence channel images as an 8‐bit type and adjusting the threshold using the ‘Analyse Particles’ features with the same parameters in Image J software (version 1.53t, NIH, Bethesda, USA).

### 2.10 Mitochondrial Morphology (Mito‐Tracker) and MMP Assays

The changes in mitochondrial morphology and mitochondrial membrane potential (MMP) induced by hypoxia exposure were assessed using Mito‐Tracker Green and JC‐1 cationic fluorescent indicator (Molecular Probes; Invitrogen, USA), which aggregates in intact mitochondria to show red fluorescence indicating high or normal MMP while remaining in monomeric form in the cytoplasm shows green fluorescence, respectively. Briefly, both OECM‐1 and OECM‐1 CSC cells (a final density of 5 **×** 10^3^ cells/mL) were seeded in triplicates in DMEM low glucose with 10% FBS in a 6‐well plate under either normoxic conditions (21% O_2_) or hypoxic conditions (1% O_2_) for variable periods (0, 6, and 16 h). After the hypoxia exposure, the medium was removed, followed by staining with Mito‐Tracker Green (100 nM final concentration) and JC‐1 dye (10.0 μM final concentration) for 30 min at 37°C in the incubator. After washing three times with 1 × PBS for 5 min, samples were mounted with fluorescence mounting media (Thermo Scientific, USA), and finally, images were captured wherein mitochondrial fragmentation and MMP were visualised using a confocal microscope (Leica TCS‐SPE Microsystems, Germany) with a 63× objective. The ratio of red (at 525 nm excitation and 590 nm emission) and green fluorescence intensity (at 490 nm excitation and 530 nm emission) from JC‐1 dye was calculated using ImageJ software (version 1.53t, NIH, Bethesda, USA).

### 2.11 Metabolism Measurements

The oxygen consumption rate (OCR) and extracellular acidification rate (ECAR) were measured using a 24‐well microplate Seahorse XF V7 flux analyser (Seahorse Bioscience, USA) as followed by previously published methods with minor variations [34]. In brief, both OECM‐1 and OECM‐1 CSC cells (final density of 2 **×** 10^5^ cells/mL) were seeded in triplicates in DMEM low glucose with 10% FBS in a 24‐well plate and then incubated for 24 h under hypoxia. On the next day, the plate was re‐incubated at 37°C for 1 h under normoxic conditions, except with no CO_2_, before analysing of the OCR and ECAR. All analyses were first normalised to the total protein content. Finally, the mitochondria OCR was calculated by subtracting the final OCR values (pmolO_2_/min) after Antimycin A, oligomycin and FCCP treatment from the average of the three OCR values before Antimycin A, oligomycin and FCCP treatment.

### 2.12 Small Interfering RNA Transfection (siRNA) for BNIP3 Silencing

Human oral squamous carcinoma OECM‐1 was transfected with two different sequences of siRNA (100 nM) targeting BNIP3 (si‐BNIP3; Ambion, Life Technologies, CA, USA) using a Lipofectamine 2000 (GIBCO, MD, USA) transfection kit according to the instructions manual. Next, the knockdown efficiency of siBNIP3 was examined by Western blot assays. For this study, we used the following BNIP3 siRNA sequences: BNIP3 siRNA‐1 (sense [5′–3′] GAAAAACUCAGAUUGGAUAtt, antisense [5′–3′] UAUCCAAUCUGAGUUUUUCtt); BNIP3 siRNA‐2 (sense [5′–3′] GGUUUCUAUUUAUAAUGGAtt, antisense [5′–3′] UCCAUUAUAAAUAGAAACCga).

### 2.13 Bioinformatics Analysis (TGCA Data Sets)

Details regarding BNIP3 and BNIP3L expression in primary tumour samples from HNSC patients were obtained from the TCGA database using the UALCAN data analysis portal (http://ualcan.path.uab.edu/) and were subsequently analysed. Next, we performed a Kaplan–Meier survival analysis for HNSC patients based on the differential expression of BNIP3.

### 2.14 Statistical Analysis

All data are expressed as mean ± SD for all the in vitro experiments with a minimum of three independent biological replicates (as in triplicate measurements). All statistical values were calculated by GraphPad Prism (version 7.0, Boston, USA), while their analysis was carried out using one‐way analysis of variance (ANOVA) Tukey multiple comparisons or two‐tailed Student's *t*‐test, unless otherwise stated, with *p* < 0.05 considered statistically significant.

## 3 Results

### 3.1 Functional Validation and Characterisation of OECM‐1 CSCs

**FIGURE 1:**
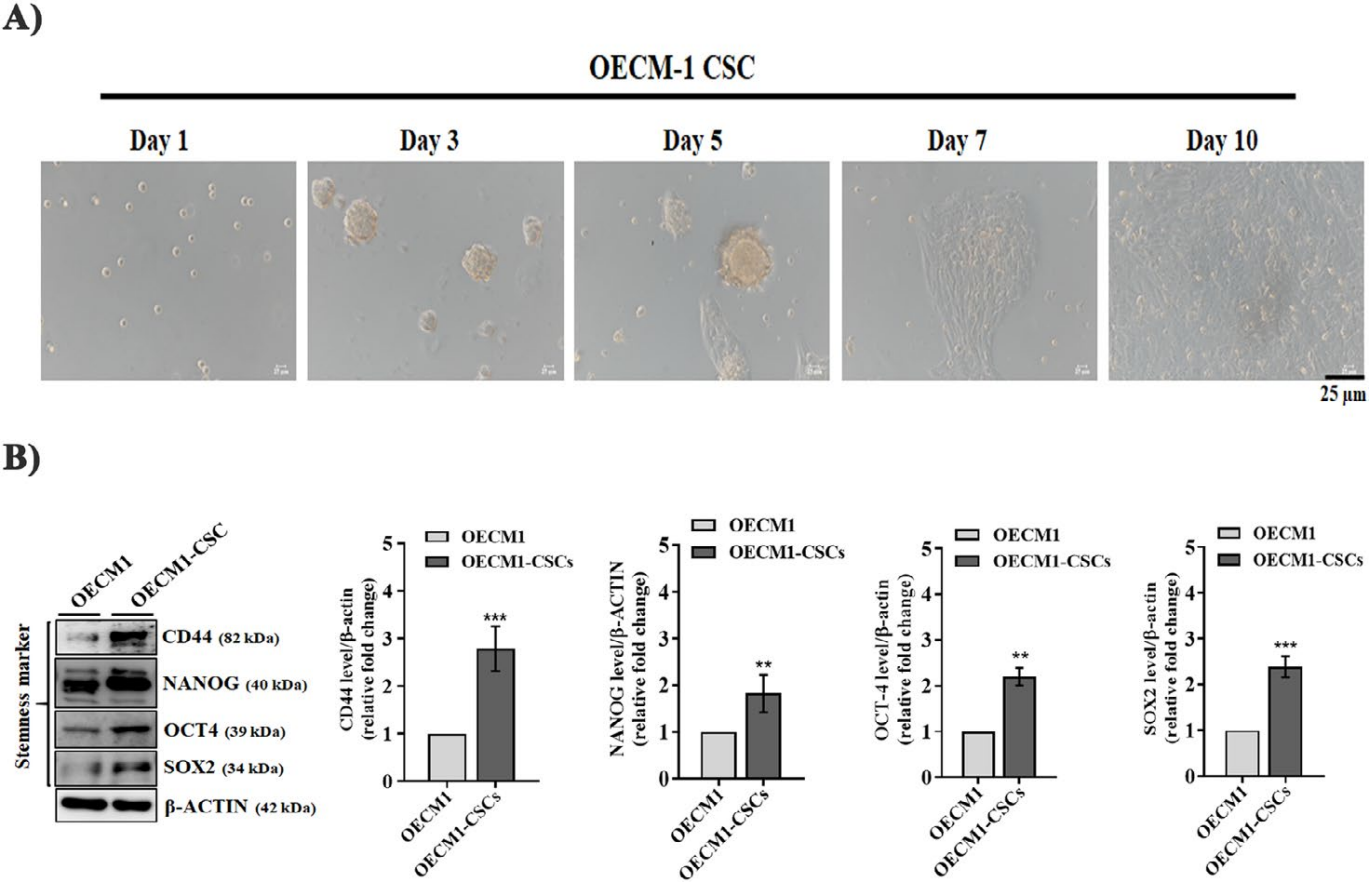
Functional validation and characterisation of OECM‐1 CSCs by cell morphology and stemness markers analysis. (A) Representative images show cell morphology and sphere‐like bodies in growth factor (EGF and bFGF) added OECM‐1 cultured cells within 10 days, wherein OECM‐1 cells transformed into stem‐like cells. Scale bar = 25.0 μm. (B) Western blotting analysis of the stemness marker protein expression (CD44, NANOG, OCT4 and SOX2) in both OECM‐1 and OECM‐1 CSCs. Data presented are mean ± SD (*n* = 3). *p < 0.05, **p < 0.01 and ***p < 0.001 versus OECM‐1 cells.

In the present study, we used OECM‐1 CSCs that were isolated from the human OECM‐1 cell lines. Here, first, we cultured OECM‐1 cells in serum‐free medium (SFM) containing bFGF and EGF. After being cultured for approximately 2 weeks, a subset of OECM‐1 cells gradually started to form spheres. Next, we confirmed the phenotype and functional characteristics of OECM‐1 CSCs, i.e., dedifferentiation, sphere formation, and stemness features. From day 1 to 10, morphological changes in OECM‐1 CSCs were observed and recorded. On day 1, OECM‐1 CSCs were flattened and dispersed, and then a few stem cell colonies appeared on day 3. Further, microscopy images showed that the CSCs gradually developed a uniform shape by day 5. On days 7 and 10, colony accumulation and sphere formation revealed a significant increase in the OECM‐1 CSCs subculture. As analysed in Figure 1A, compared to the other days, day 10 displayed the most sphere formation. To further confirm the characteristics of OECM‐1 CSCs, we examined the expression of stemness genes (NANOG, SOX2, CD44, and OCT4) by western blot assay. As shown in Figure 1B, the protein levels of NANOG, SOX2, CD44, and OCT4 significantly increased with time, with the highest levels recorded on day 10 of OECM‐1 CSCs compared to OECM‐1 cells. These results demonstrate that OECM‐1 CSCs may have stronger stemness properties, spheroid formation efficiency, and spontaneously differentiate towards the epithelium in the long‐term subculture.

### 3.2 Effects of Hypoxia Exposure on the Cell Viability of OECM‐1 and OECM‐1 CSCs

**FIGURE 2:**
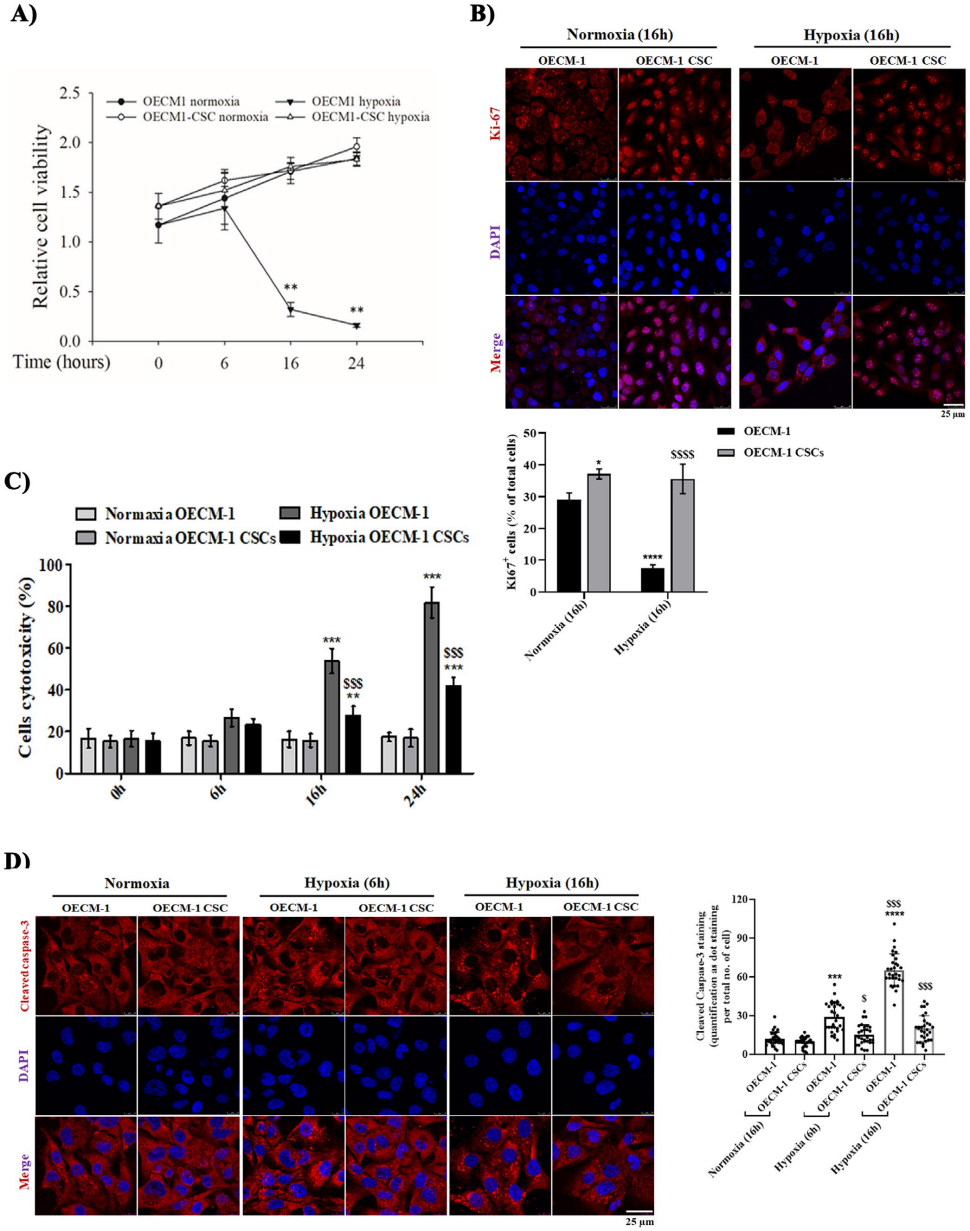
OECM‐1 CSC have higher survival rates than OECM‐1 under hypoxia exposure. (A) OECM‐1 and OECM‐1 CSCs were exposed to hypoxia for different time periods (0, 6, 16 and 24 h) and examined for cell viability using the CCK8 assay. Significance is ascribed as **p < 0.01 versus OECM‐1 and OECM‐1 CSC control (normoxia). (B) Representative confocal images of hypoxia‐exposed OECM‐1 and OECM‐1 CSCs immunostained with anti‐Ki‐67 (a prominent marker of proliferation). The quantification of Ki67^+^ staining data is represented as the count of Ki67^+^ cells per total number of cells. Scale bar = 25.0 μm. (C) Cell cytotoxicity was measured in hypoxia‐treated OECM‐1 and OECM‐1 CSCs after 6, 16 and 24 h using the LDH cytotoxicity assay. The data for cell viability and cytotoxicity are presented as percentages and fold change, respectively, and statistically described as mean ± SD. (D) Representative confocal images of cleaved caspase‐3 immunostaining in both normoxic and hypoxic OECM‐1 and OECM‐1 CSCs for 0, 6 and 16 h. Scale bar = 25.0 μm. Statistically, the data are presented as mean ± SD. Significance is ascribed as *p < 0.5, **p < 0.01 and ***p < 0.001 versus OECM‐1 and OECM‐1 CSC control (normoxia); ^$^ *p* < 0.05, ^$$^ *p* < 0.01, ^$$$^ *p* < 0.001 and *p* < 0.0001 versus hypoxia‐exposed OECM‐1 cells. These experiments were independently carried out more than three times.

To investigate the effects of hypoxia treatments on cell viability and cytotoxicity of both OECM‐1 and OECM‐1 CSCs, we first examined cell viability using the CCK‐8 assay. The CCK‐8 assay results showed that cell viability significantly decreased in OECM‐1 cells in a time‐dependent manner after hypoxia exposure (6, 16 and 24 h) compared to their normoxia conditions (Figure 2A); however, OECM‐1 CSCs didn't show significant differences compared to similar cells harvested in normoxia. Furthermore, cancer cell proliferation was examined using immunochemical staining of Ki67 (a prominent cell proliferation marker). Compared with the OECM‐1 cells after 16 h of hypoxia exposure, OECM1‐CSCs displayed significantly increased Ki67^+^ staining, with most of the cells showing Ki67^+^ among the total number of viable cells (Figure 2B). However, the percentage of Ki67^+^ cells in OECM‐1 CSCs under both normoxia and hypoxia did not show a major difference between these two groups (as almost all Ki67^+^ cells in OECM‐1 CSCs under both normoxia and hypoxia were positive for Ki67^+^). These results are consistent with the cell viability data from the CCK‐8 assay. Collectively, the above results demonstrate that OECM‐1 CSCs have higher cancer cell proliferation and hypoxia‐resistant properties, indicating that CSCs are required for establishing more tumour cell proliferation features under hypoxic stress environments.

Further, we assessed cytotoxicity using an LDH cytotoxicity assay. We observed that higher cytotoxicity was found in OECM‐1 cells under similar hypoxia exposure than in cells cultivated under normoxia conditions, and there was no significant difference between OECM‐1 CSCs and their normoxia counterparts (Figure 2C). In addition, cell death was also determined by detection of cleaved effector Caspase‐3 immunostaining, wherein cleaved caspase‐3 immunostaining was significantly increased in OECM‐1 cells at 6 and 16 h of hypoxiaexposure compared to that in the cells under normoxia exposure, and the same was significantly decreased in the OECM‐1 CSCs under similar hypoxia exposure (Figure 2D). These results indicate that OECM‐1 CSCs exhibit higher proliferation efficiency and sphere formation phenotype due to their resistance to hypoxia‐induced apoptosis.

### 3.3 Effects of Hypoxia Exposure on the Cell Migration and Invasion Capabilities of OECM‐1 and OECM‐1 CSCs

**FIGURE 3:**
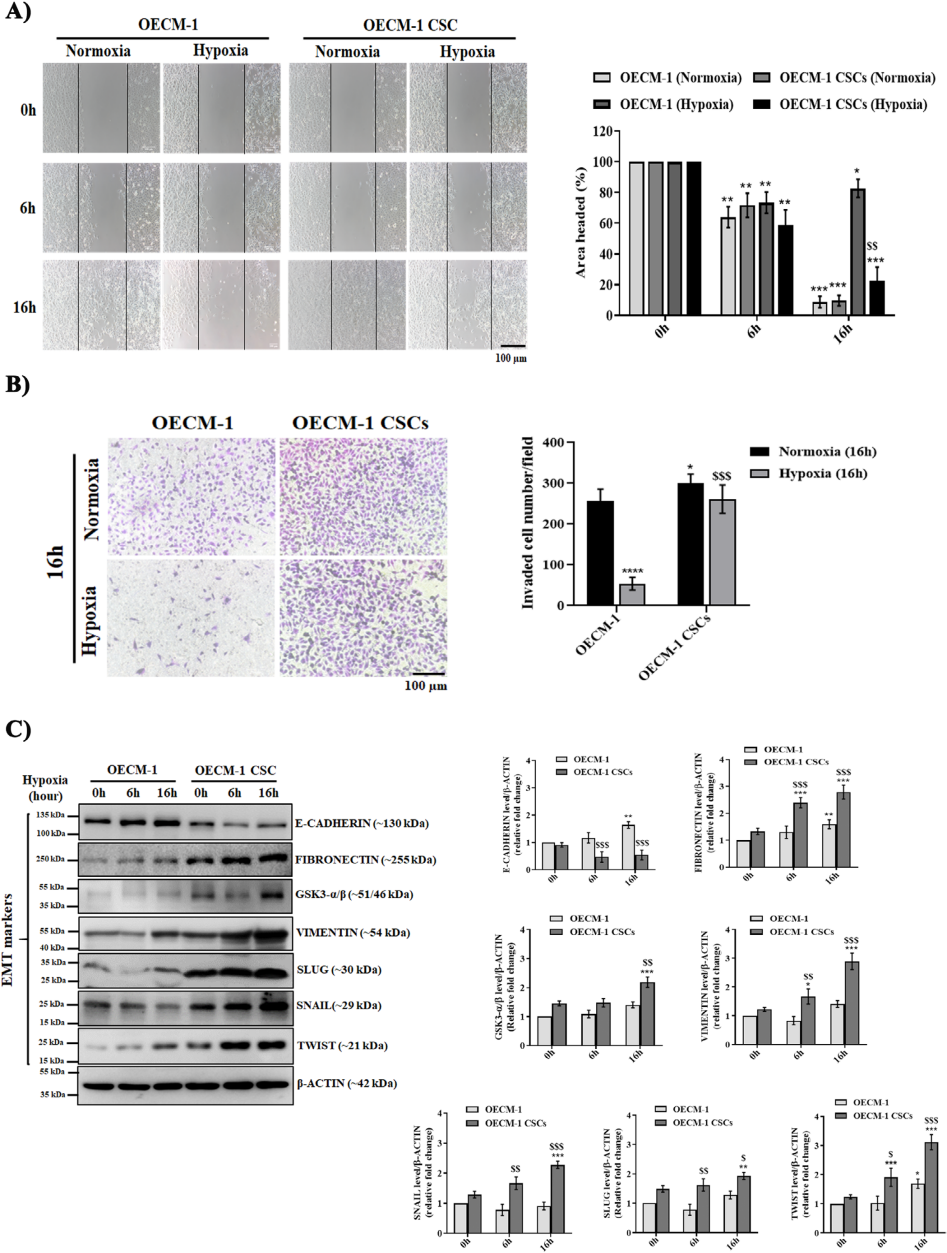
OECM‐1 CSCs exhibit higher migration and invasion capacity and EMT‐like phenotypes than OECM1 under hypoxia. (A) Representative microscopic images of the wound healing assay of 16 h exposed OECM‐1 and OECM‐1 CSCs under normoxic and hypoxic conditions. Scale bar = 100.0 μm. The graphs show the percentage of area covered. (B) Representative microscopic images of the invasion assay in normoxic and 16 h hypoxia‐exposed OECM‐1 and OECM‐1 CSCs. Scale bar = 100.0 μm. Graphs show the average number of invaded cells per field. (C) The expression of EMT‐marker proteins in both normoxic and hypoxic OECM‐1 and OECM‐1 CSCs was examined using western blot analysis with β‐Actin as the endogenous loading control. Data are presented as mean ± SD. Significance is ascribed as *p < 0.5, **p < 0.01 and ***p < 0.001 versus OECM‐1 and OECM‐1 CSCs control (normoxia); ^$^ *p* < 0.5, ^$$^ *p* < 0.01 and ^$$$^ *p* < 0.01 versus hypoxia‐exposed OECM‐1 cells. These data were carried out independently more than three times.

To examine how hypoxia pre‐exposure influenced the biological behaviours of the OECM‐1 and OECM‐1 CSCs, we carried out wound healing and matrigel assays for migration and invasion, respectively. In the wound healing assay, the scratch wound area was significantly smaller in OECM‐1 CSCs than in OECM‐1 cells after exposure to hypoxia. As shown in Figure 3A, the migratory capability of the OECM‐1 CSCs was significantly higher by approximately 21% and 36% compared to that of OECM‐1 cells after 6 and 16 h hypoxia exposure, respectively. Moreover, our invasive assay analysis revealed a significantly lower number of invasive cells in 16 h hypoxia‐pretreated OECM‐1 cells. As shown in Figure 3B, there is a ~48% reduction in the number of invasive cells in the OECM‐1 cells under 16 h hypoxia exposure compared to OECM‐1 CSCs under a similar exposure regimen. The results suggested that hypoxia‐exposed OECM‐1 CSCs have enhanced migration and invasive capability compared to OECM‐1 cells.

### 3.4 OECM‐1 CSCs Maintain EMT‐Like Phenotypes Under Hypoxia Exposure

Earlier studies reported that higher EMT plays a significant role in the acquisition of cancer stemness under the hypoxic microenvironment as the leading cause of invasion and metastasis. To examine this, the expression of numerous EMT markers was analysed in the OECM‐1 and OECM‐1 CSCs under hypoxia exposure using the Western blot assay. As shown in Figure 3C, the protein expression of FIBRONECTIN, GSK3‐α/β, VIMENTIN, SNAIL, SLUG, and TWIST was significantly increased in hypoxia‐treated (6 and 16 h) OECM‐1 CSCs compared to similarly exposed OECM‐1 cells. However, the expression of E‐CADHERIN was significantly decreased in OECM‐1 CSCs compared to in OECM‐1 cells under similar hypoxia exposure. Thus, the switching of E‐CADHERIN expression indicates that the EMT‐like property has occurred under hypoxic conditions in the OECM‐1 CSCs.

### 3.5 OECM‐1 CSCs Resistant to Hypoxia Induce Higher ROS and Mitochondrial Dysfunctions

**FIGURE 4:**
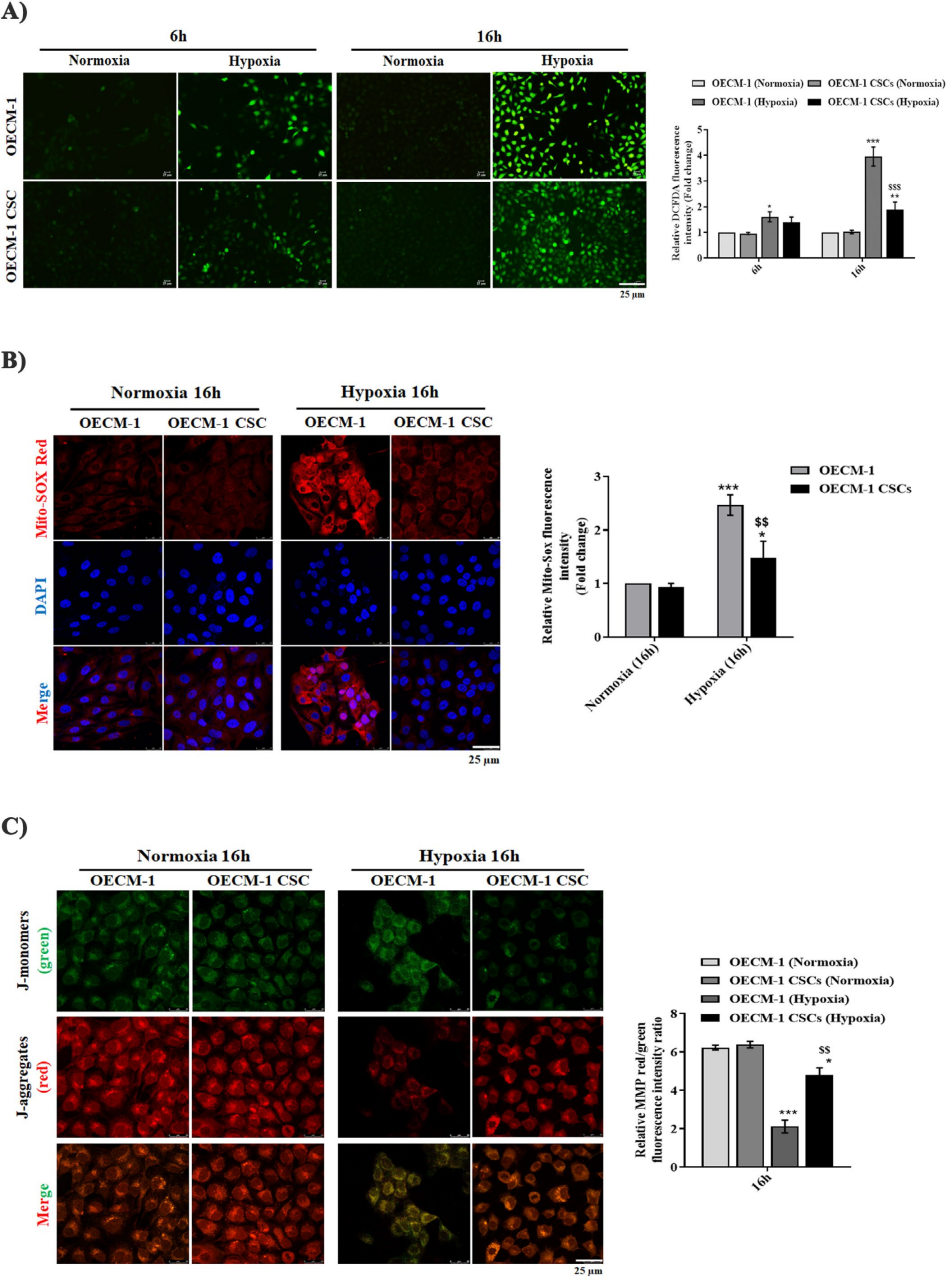
Lower levels of ROS and mitochondrial dysfunction in hypoxia‐exposed OECM‐1 CSCs. Representative confocal microscopic images showing (A) DCFDA (as an indicator of general ROS level) (scale bar = 25.0 μm) and (B) Mito‐SOX (as an indicator of mitochondrial $O_{2}^{∙-}$ level) measured in OECM‐1 and OECM‐1 CSCs cultured under normoxic or hypoxic conditions for 6 and 16 h. Scale bar = 25.0 μm. (C) Representative images of mitochondrial membrane potential measured by JC‐1 fluorescence dye (aggregates, red; monomers, green) in normoxia‐ and hypoxia‐exposed OECM‐1 and OECM‐1 CSCs for 16 h. Scale bar = 25.0 μm. Data are presented as mean ± SD and obtained from more than three independent experiments. Significance is ascribed as *p < 0.5 and ***p < 0.001 versus OECM‐1 and OECM‐1 CSCs control (normoxia); ^$$^ *p* < 0.01 and ^$$$^ *p* < 0.001 versus hypoxia‐exposed OECM‐1 cells.

**FIGURE 5:**
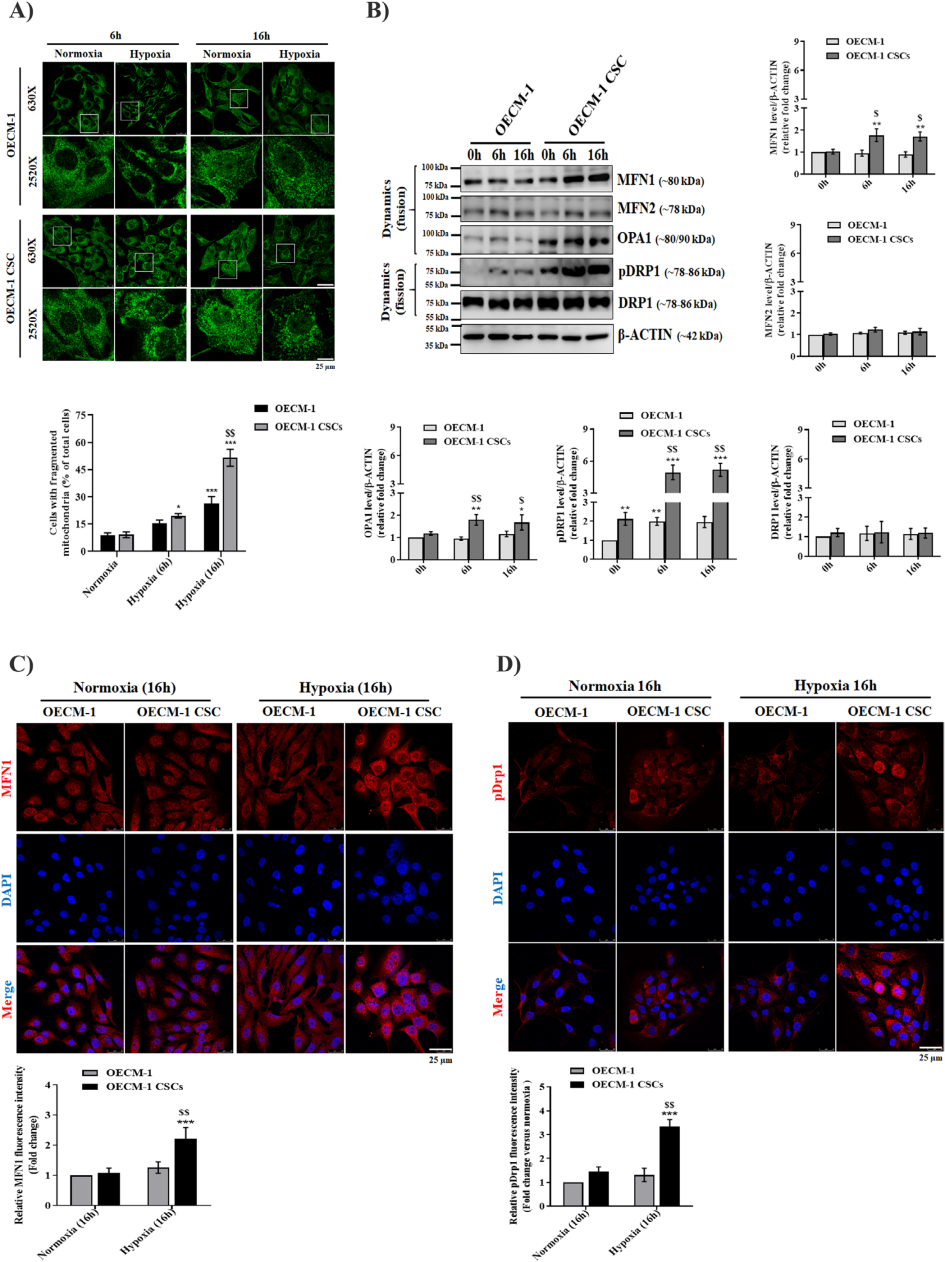
OECM‐1 CSCs exhibit an enhanced rate of mitochondrial fission compared to OECM‐1 cells under hypoxia. (A) Representative confocal microscopic images show the mitochondrial morphology examined by mitotracker staining in OECM‐1 and OECM‐1 CSCs cultured either in normoxia or a hypoxia chamber for 6 and 16 h. Scale bar, 25.0 μm. (B) Western blot analysis shows levels of the mitochondrial fusion (MFN1, MFN2, and OPA1) and fission (pDRP1 and DRP1) proteins in normoxia‐ and hypoxia‐exposed OECM‐1 and OECM‐1 CSCs for 6 and 16 h. Immunofluorescence images of DRP1 (C) and MFN1 (D) in cultured cells of OECM‐1 and OECM‐1 CSCs either exposed to normoxia or hypoxia for 16 h (scale bar, 25.0 μm). Data are presented as mean ± SD and performed independently more than three times. Significance is ascribed as *p < 0.5, **p < 0.01 and ***p < 0.001 versus OECM‐1 and OECM‐1 CSCs control (normoxia); ^$^ *p* < 0.05 and ^$$^ *p* < 0.01 versus hypoxia‐exposed OECM‐1 cells.

To investigate the role of mitochondrial homeostasis in the OECM‐1 and OECM‐1 CSCs' proliferation and stemness function under hypoxic conditions, we first examined the intracellular ROS and mitochondrial superoxide production using a DCFDA (a general indicator of ^•^OH radical formation) green and Mito‐SOX (a general indicator of $O_{2}^{∙-}$ formation) red immunostaining assay. As shown in the Figure 4A,B, higher fluorescence intensity of both intracellular and mitochondrial‐derived ROS was observed in the OECM‐1 cells after 6 h and 16 h hypoxia treatment than in OECM‐1 CSCs under similar exposure conditions. Furthermore, we examined mitochondrial membrane potential (MMP) using JC1 dye staining and found that hypoxia (6 and 16 h) treated OECM‐1 cells exhibited a lower MMP, evidenced by fluorescence transition from red to green indicating the loss of MMP and mitochondrial damage, compared with OECM‐1 CSCs under similar exposure conditions (Figure 4C). Next, we examined mitochondrial morphology using Mito tracker Green fluorescence staining. We found a significant increase in mitochondrial fragmentation, evidenced by small, round, or dot‐like staining patterns, in 6 and 16 h hypoxia‐treated OECM‐1 CSCs cells compared to similarly treated OECM‐1 cells (Figure 5A). These results further prompted us to examine the expression of mitochondrial dynamics proteins (fusion and fission) in the OECM‐1 and OECM‐1 CSCs after hypoxia exposure. Interestingly, we found a significant increase in the expression of mitochondrial fusion proteins, including mitofusin 1 and 2 (MFN1/2) and optic atrophy (OPA), in OECM‐1 CSCs after 6 and 16 h hypoxia exposure compared with similarly treated OECM‐1 cells. Moreover, we identified the expression of both total and activated DRP1 (pDRP1 Ser616), a prominent marker of mitochondrial fission, in OECM‐1 and OECM‐1 CSCs after hypoxia exposure. Interestingly, we found that the expression of pDrp1 was significantly increased in OECM‐1 CSCs compared to OECM‐1 cells after 6 and 16 h of hypoxia exposure relative to their respective cells cultured in normoxic conditions (Figure 5B). A similar trend was observed when pDRP1 immunostaining was used to examine mitochondrial fission in OECM‐1 and OECM‐1 CSCs under similar hypoxic treatment (Figure 5C). These data suggest that higher DRP1 was phosphorylated by hypoxia treatment, which indicates that mitochondrial dysfunction occurs in the cancer cells. Further, considering earlier findings, we suggested that higher mitochondrial fragmentation could aid in the segregation of dysfunctional mitochondria and their elimination through mitophagy, which can lead to increased cancer cell growth and tumour progression.

**FIGURE 6:**
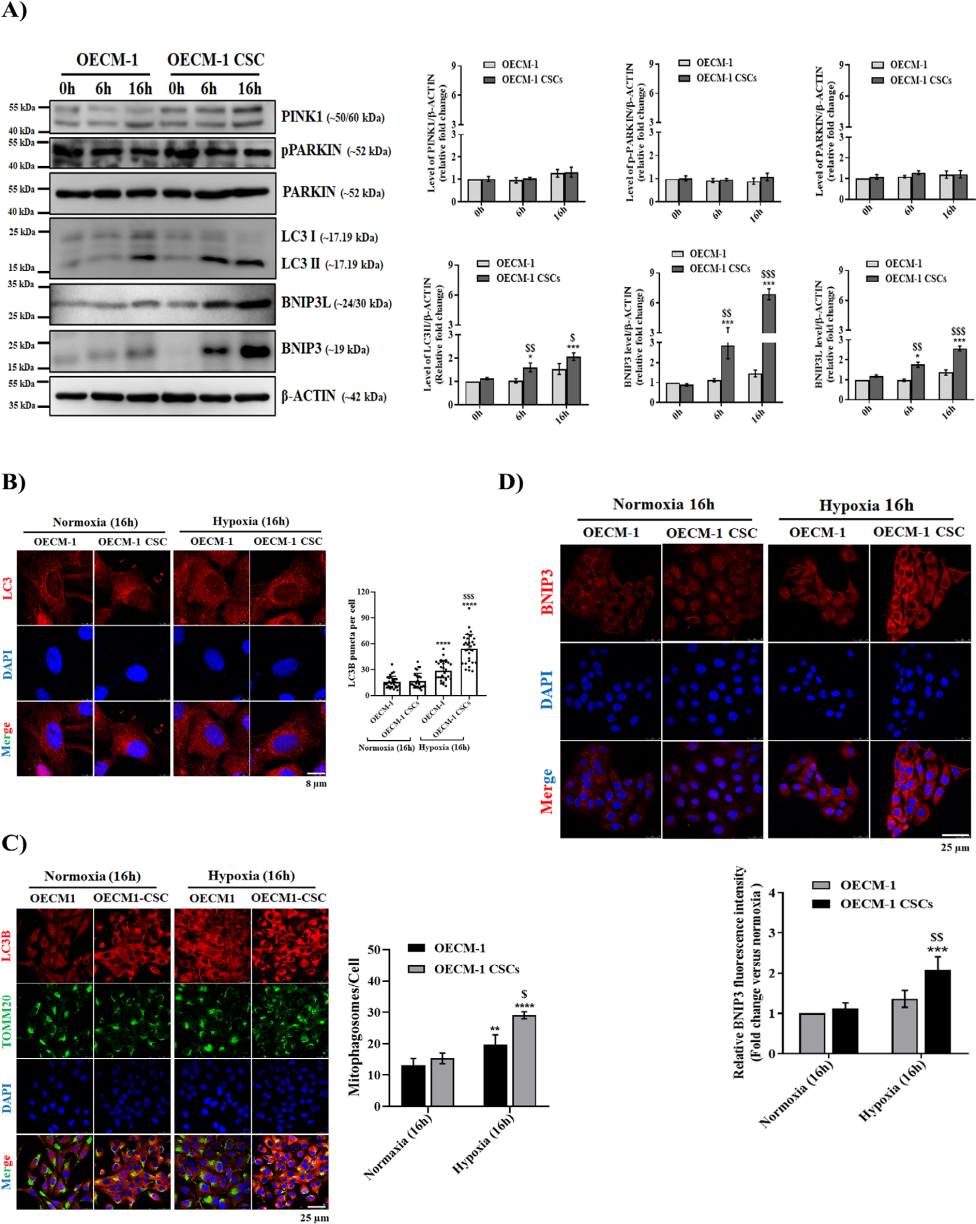
Hypoxia‐exposed OECM‐1 CSCs exhibit enhanced levels of mitophagy and autophagy. (A) Western blot analysis showing levels of the mitophagy/autophagy markers (PINK1, PARKIN, pPARKIN, LC3I/II, BNIP3, and BNIP3L) in the whole‐cell lysates of normoxia‐ and hypoxia‐exposed OECM‐1 and OECM‐1 CSCs for 6 and 16 h. (B) Representative immunofluorescence images of LC3B staining (quantification presented as number of LC3B puncta/cell) (scale bar, 8.0 μm), co‐localised LC3B and TOMM20 (C), and BNIP3 (D) in cultured cells of OECM‐1 and OECM‐1 CSCs that were either exposed to normoxia or hypoxia for 16 h (scale bar, 25.0 μm). Values are mean ± SD, with replicates performed independently more than three times. Significance is ascribed as *p < 0.5, **p < 0.01, ***p < 0.01 and ****p < 0.0001 versus OECM‐1 and OECM‐1 CSCs control (normoxia); ^$^ *p* < 0.05, ^$$^ *p* < 0.01 and ^$$$^ *p* < 0.01 versus hypoxia‐exposed OECM‐1 cells.

To clarify whether hypoxia‐induced mitochondrial dysfunctions can induce mitophagy in the OECM‐1 and OECM‐1 CSCs, we performed a mitophagy analysis using western blot and immunofluorescence staining assays. Earlier studies reported that mitophagy is one of the important ways to determine mitochondrial homeostasis and plays a key role in maintaining the stemness features of cancer cells [24, 35]. As shown in Figure 6A, compared with OECM‐1 cells, the levels of LC3B (a marker of autophagosome complex formation) in the whole‐cell lysate were significantly increased in OECM‐1 CSCs after 6 and 16 h of hypoxia treatment; a similar trend was also observed with the LC3B fluorescence staining in terms of the number of LC3B puncta (Figure 6B), further confirming that higher autophagy induction occurs in OECM‐1 CSCs under hypoxic stress compared to OECM‐1 cells under a similar exposure regimen. Furthermore, we quantified “mitophagosome” by colocalizing LC3‐positive puncta and a mitochondrial outer membrane protein, TOMM20. Compared with OECM‐1 cells, we observed a significant increase in mitophagosome formation in OECM‐1 CSCs under 6 and 16 h of hypoxia treatment, indicative of active mitophagy in OECM‐1 CSCs under hypoxia (Figure 6C). We then investigated the effect of hypoxia on receptor‐mediated mitophagy BNIP3/‐L, which functions as a requirement for autophagic machinery (LC3, p62 and Beclin1) at the surface of mitochondria. It was observed that the levels of BNIP3 and BNIP3L significantly increased in the OECM‐1 CSCs after 6 and 16 h of hypoxia treatment compared to similarly exposed OECM‐1 cells; a similar trend was also observed with BNIP3 fluorescence staining (Figure 6A,D). Numerous studies have reported that crosstalk exists between receptor‐ and non‐receptor‐mediated pathways to fine‐tune mitophagy. To understand the changes in non‐receptor‐mediated pathways during hypoxia‐induced oral cancer cell mitophagy, unlike BNIP3, the levels of PINK1, pPARKIN and PARKIN proteins were slightly increased in OECM‐1 CSCs after 6 and 16 h of hypoxia exposure compared to OECM‐1 cells. However, a comparison among OECM‐1 and OECM‐1 CSCs revealed an insignificant change in the levels of PINK1, pPARKIN, and PARKIN proteins under similar hypoxia exposure (Figure 6A). These results indicate that hypoxia‐induced BNIP3/‐L‐driven mitophagy could participate in the activation of mitophagy/autophagy pathways in OECM‐1 CSCs under hypoxic conditions.

### 3.6 Mitochondrial Metabolism Reprogramming Regulates the Cancer Stemness Features of Hypoxia‐Treated OECM‐1 CSCs

**FIGURE 7:**
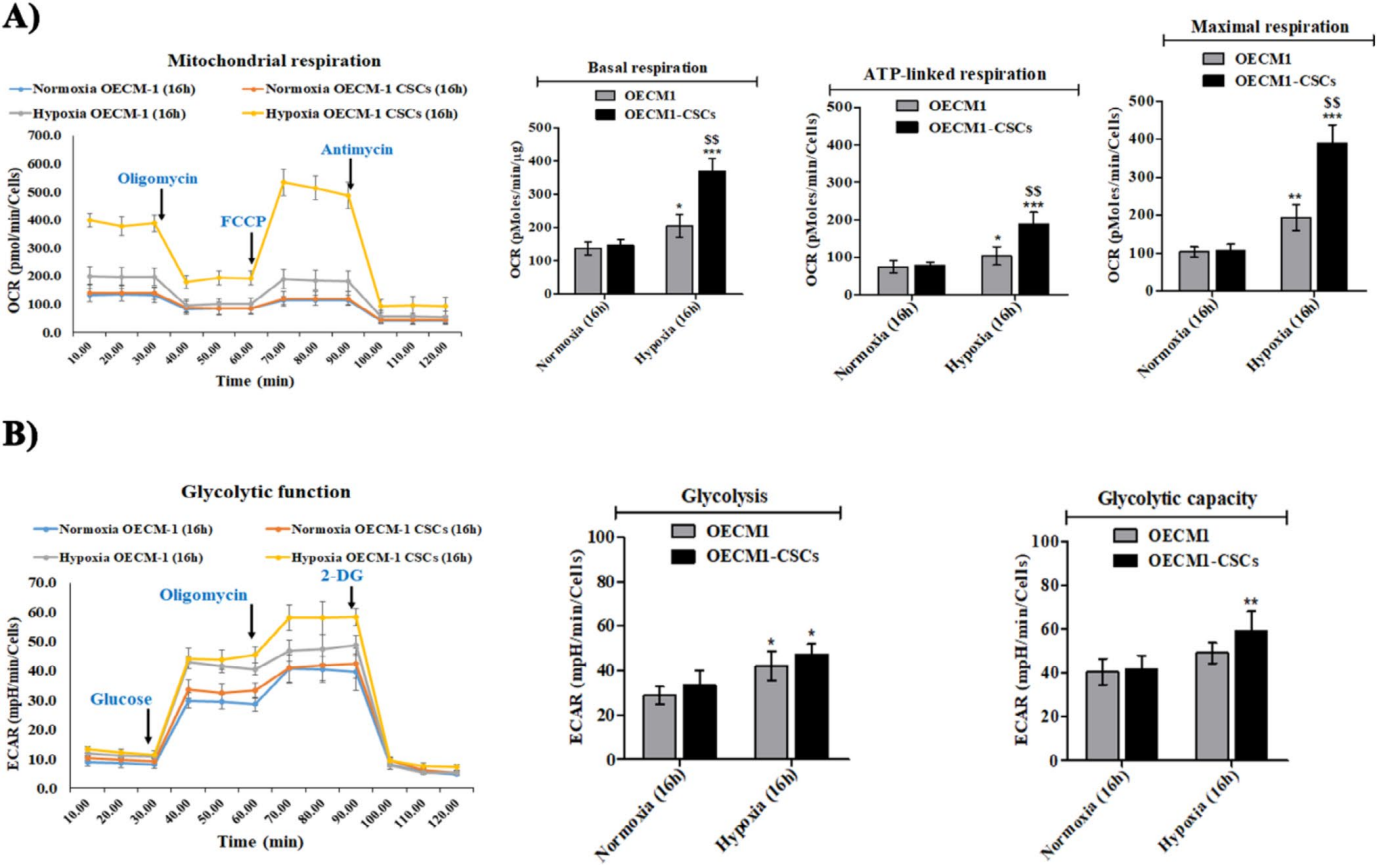
Hypoxia‐exposed OECM‐1 CSCs exhibit increased mitochondrial metabolism reprogramming. (A) The oxygen consumption rate (OCR), an indicator of mitochondrial OXPHOS and (B) The extracellular acidification rate (ECAR), an indicator of glycolytic metabolism were measured using the Seahorse 96 XF analyser in normoxia‐ and hypoxia‐exposed OECM‐1 and OECM‐1 CSCs, normalised per μg of protein. Both OECM‐1 and OECM‐1 CSC cells were injected at indicated times with oligomycin (ATP synthase inhibitor), FCCP, rotenone, 2‐DG, glucose, and antimycin. Values are mean ± SD and were performed in independent replicates more than three times. Significance is ascribed as *p < 0.5, **p < 0.01 and ***p < 0.001 versus OECM‐1 and OECM‐1 CSC control (normoxia); ^$$^ *p* < 0.01 versus hypoxia‐exposed OECM‐1 cells.

Mitochondria perform cellular energetic metabolism and are regulated through a balance between mitochondrial dynamic functions (fusion and fission). Earlier studies indicate increased mitochondrial fission aggravates the cancer cells' metabolism rates and, as a result, increases migration and invasion in numerous cancers. To elucidate whether hypoxia treatment leads to changes in the energetic metabolisms in the OECM‐1 and OECM‐1 CSCs, we next used a Seahorse XF analyser to determine the oxygen consumption rate (OCR) in tumour cells, a marker of oxidative metabolism. We observed a significant increase in respiration capacity, including both basal and maximal respiration, in OECM‐1 CSCs compared with OECM‐1 cells under 16 h hypoxia exposure. Intriguingly, OECM‐1 CSCs also exhibited a significant increase in ATP production compared to OECM‐1 cells under similar hypoxia conditions (Figure 7A). Further, we examined the ECAR rate as a representative of glycolytic activity, which was mildly increased in OECM‐1 CSC cells as compared to the OECM‐1 cells after 16 h of hypoxia (Figure 7B), highlighting that mitochondrial OXPHOS was significantly enriched in OECM‐1 CSCs. Together, these results suggest that hypoxia induces mitochondrial OCR metabolism reprogramming and may function as a general determinant of cancer stem‐like properties.

### 3.7 BNIP3/L‐Dependent Mitophagy Regulates the Cancer Stemness Feature of Hypoxia‐Treated OECM‐1 CSC

**FIGURE 8:**
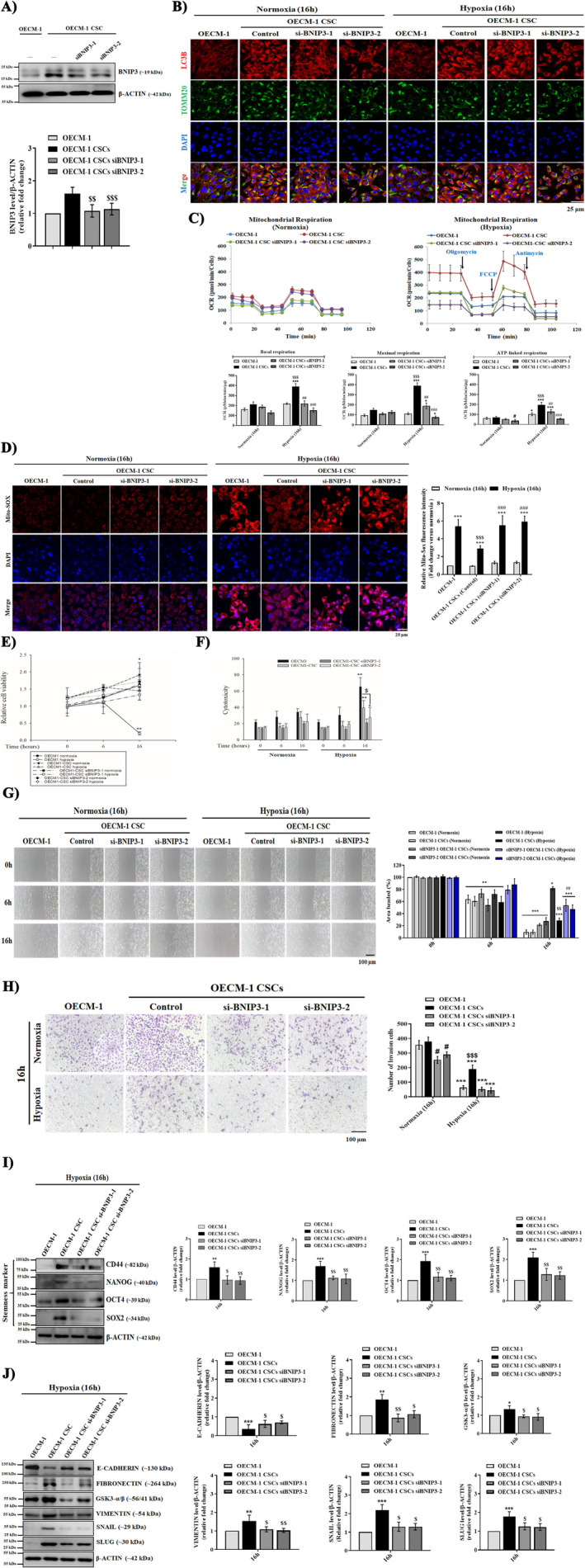
BNIP3 enhanced the migration and invasion properties and suppressed mitochondrial dysfunction in OECM‐1 CSCs under hypoxic conditions that promote stemness capacity. To elucidate whether BNIP3/‐L‐induced autophagy was involved in the stemness functions of oral CSCs under hypoxia exposure, siRNA‐targeting BNIP3 (siBNIP3) was used to silence BNIP3 expression. (A) Western blots were performed to examine the effects of siRNA on BNIP3 expression, and densitometry analysis graphs represent the band signal intensity as a fold change. (B) Representative confocal microscope images showing the localisation of LC3 in OECM‐1, OECM‐1 CSCs, siBNIP3‐1 and siBNIP3‐2 OECM‐1 CSCs under both normoxic and hypoxic conditions using TOMM20 (a receptor for proteins targeted to the outer mitochondrial membrane) staining along with graphs showing fluorescence integrated density in the OECM‐1, OECM‐1 CSCs, siBNIP3‐1 and siBNIP3‐2 OECM‐1 CSCs. Scale bar = 25.0 μm. (C) A 96 XF‐Seahorse analyser assay was performed to measure the OCR rate in the aforementioned cells under normoxic and hypoxic conditions. Representative graphs quantifying the rates of basal respiration, ATP‐linked respiration, and maximal respiration are shown. (D) MitoSOX (5 μM) was used to measure mitochondrial $O_{2}^{∙-}$ production and the graph quantifies the fluorescent intensity of MitoSOX as fold change in the OECM‐1, OECM‐1 CSCs, siBNIP3‐1, and siBNIP3‐2 OECM‐1 CSCs under both normoxic and hypoxic conditions. Scale bar = 25.0 μm. (E, F) The effect of BNIP3 silencing on cell proliferation and cytotoxicity was determined using CCK8 and LDH assays, respectively, with representative graphs quantifying the rate of cell viability and cytotoxicity of OECM‐1, OECM‐1 CSCs, and siBNIP3‐infected OECM‐1 CSCs under both normoxic and hypoxic conditions after 16 h treatment. (G) A scratch wound healing assay was applied to examine the migration of the indicated cells, and a graph quantifying the rate of migrating cells is provided. Scale bar = 100.0 μm. Rates are expressed as fold change. (H) Representative images of invasion in the aforementioned ed. cells and a graph quantifying the number of invading cells are shown. Scale bar = 100.0 μm. Protein expressions of stemness markers CD44, OCT4, NANOG, and SOX2 (I) and EMT markers E‐CADHERIN, FIBRONECTIN, GSK3‐α/β, VIMENTIN, SNAIL, and SLUG (J) were determined using Western blot assays in the indicated cells. Data are represented as mean ± SD and performed with more than three independent replicates. Significance is ascribed as *p < 0.5, **p < 0.01 and ***p < 0.001 versus OECM‐1 and OECM‐1 CSCs control (normoxia); ^$^ *p*<0.05, ^$$^ *p* < 0.01 and ^$$$^ *p*<0.001 versus hypoxia‐exposed OECM‐1 cells; ^#^ *p* < 0.05 and ^##^ *p* < 0.01 versus hypoxia‐exposed OECM‐1 CSCs.

To confirm the role of BNIP3‐driven mitophagy in oral CSC maintenance and survival under stress environments such as hypoxia, we transiently knock down BNIP3 expression in the OECM‐1 CSCs using lentiviral‐mediated siBNIP3 transfection. First, we analysed the expression of BNIP3 in siBNIP3‐1 and 2 transfected OECM‐1 CSCs. We found that the BNIP3 level significantly decreased in both siBNIP3‐1 and 2 transfected OECM‐1 CSCs compared with normal OECM‐1 CSCs and OECM‐1 cells (Figure 8A). Consistently, autophagy was also suppressed under this condition, as observed by reduced fluorescence intensity of membrane‐bound LC3 (Figure 8B). Moreover, suppression of mitophagy abolished the increase in oxygen consumption rates (basal respiration, maximum respiration, and ATP production) in siBNIP3‐transfected OECM‐1 CSC compared to OECM‐1 CSCs under both 6 and 16 h hypoxia (Figure 8C), but it had little effect on the ECAR rates (data not shown). These results indicate that BNIP3‐driven mitophagy positively regulates mitochondrial OXPHOS metabolism in oral CSCs under similar hypoxic conditions. Similarly, in siBNIP3 OECM‐1‐CSCs, the fluorescence intensity of mitochondria ROS ($O_{2}^{∙-}$ formation) was also higher than that in OECM‐1 CSCs under a similar hypoxic environment (Figure 8D), suggesting that BNIP3‐induced mitophagy plays a positive role in contributing to the increase and maintenance of mitochondrial functions in oral CSCs. Furthermore, we examined whether mitophagy suppression affected oral CSCs proliferation and metastatic features. Interestingly, we found that cell proliferation and cytotoxicity were insignificantly changed between siBNIP3 OECM‐1 CSCs and OECM‐1 CSCs under 16 h hypoxia (Figure 8E,F), while cancer stemness‐related properties such as cell migration and cancer stemness (Figure 8G,H) and EMT‐related protein markers (Figure 8I,J), measured by the wound healing and western blot assays, respectively, were significantly reduced in siBNIP3 transfected OECM‐1 CSCs compared with OECM‐1 CSCs under similar hypoxia exposure. These results indicate that the BNIP3‐driven mitophagy process plays a critical role exclusively in maintaining cancer stemness‐like properties such as invasion and metastasis under hypoxic stress.

**FIGURE 9:**
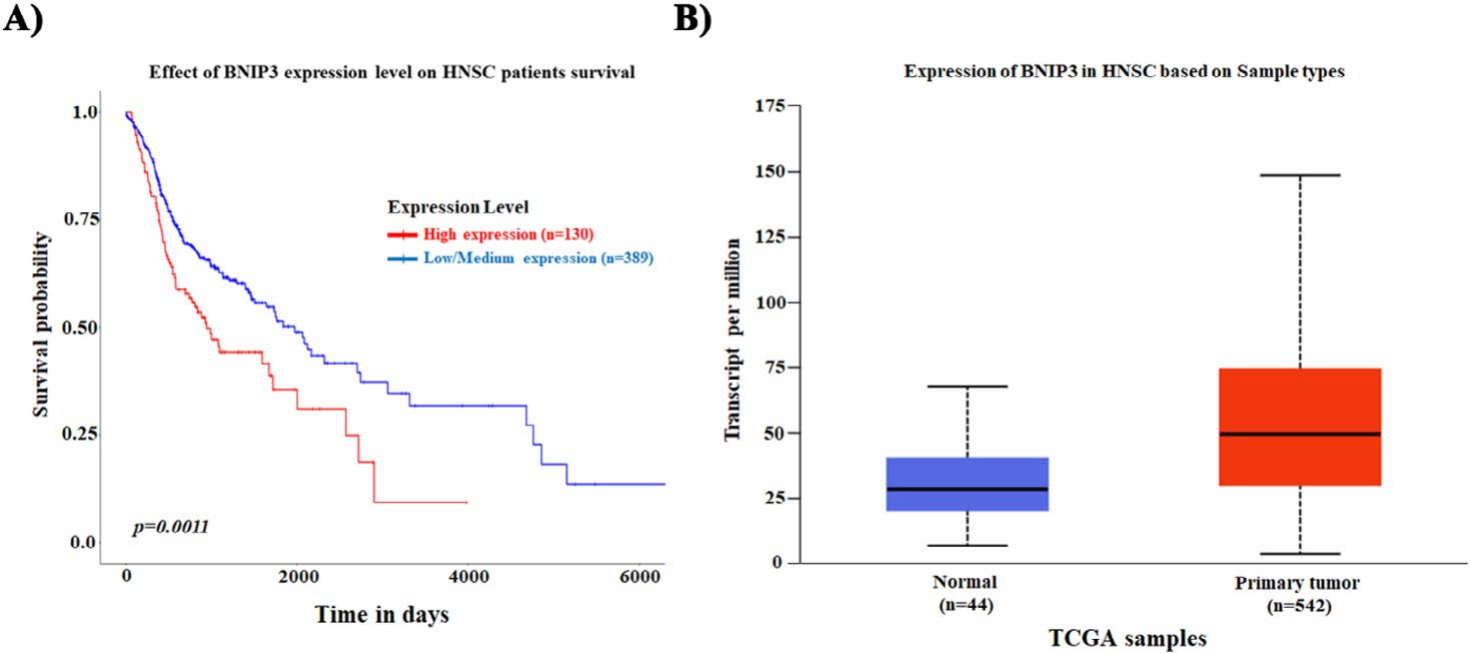
BNIP3 expression was significantly upregulated in cancers, including head and neck squamous carcinoma (HNSC). (A) The TCGA database showed that BNIP3 expression was upregulated in HNSC primary tumour samples compared to normal tissue samples. (B) Survival analysis using the HNSC Kaplan–Meier Plotter revealed that patients with higher BNIP3 expression are associated with poorer overall survival.

To further strengthen our current study conclusion, we conducted the cancer genome atlas (TCGA) data analysis of BNIP3 expression processed from the UALCAN project consortium (http://ualcan.path.uab.edu) [36]. The association of BNIP3 with Kaplan–Meier survival analysis was performed on both the publicly available cancer clinical database and the TCGA datasets. UALCAN assessed BNIP3 expression in patients with head and neck squamous cell carcinoma. The expression of BNIP3 in primary tumour tissues was significantly higher than in normal tissues (Figure 9B). Furthermore, high expression of BNIP3 was associated with worse survival in patients with HNSC (Figure 9A).

## 4 Discussion

**FIGURE 10:**
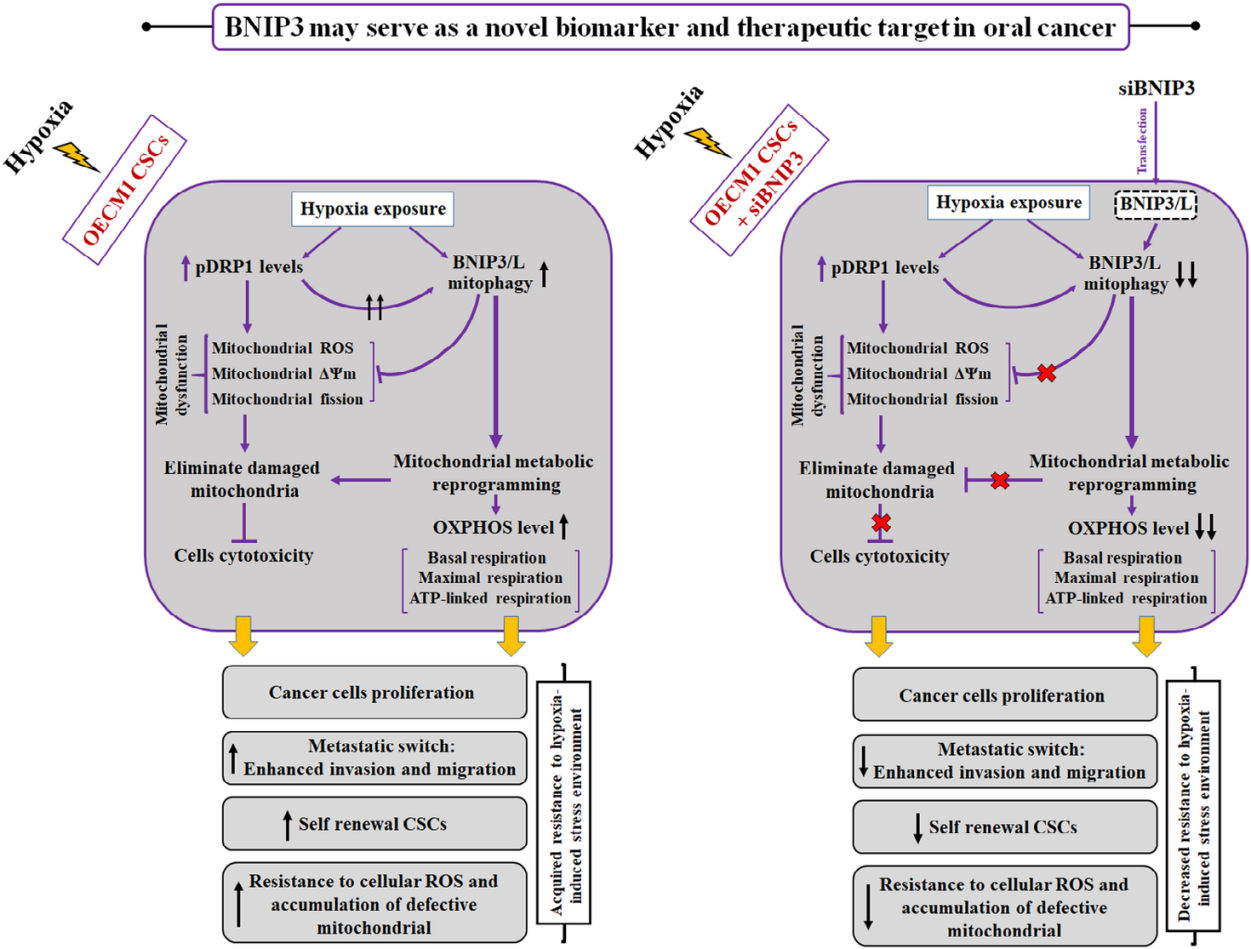
Schematic diagram depicting the role of BNIP3 in maintaining oral CSCs and their survival under hypoxic stress conditions.

In this study, we showed that increased BNIP3‐driven mitophagy in hypoxia‐exposed oral CSCs, mainly via activation of DRP1 (higher phosphorylation at Ser616), resulted in cancer cell growth, including increased cell proliferation, reprogrammed glycometabolism to OXPHOS, and EMT. In addition, UALCAN TCGA project analysis revealed that higher BNIP3 expression in HNSC primary tumour samples correlated with decreased patient survival. Further analysis indicated that the aforementioned changes in the normal functions of CSCs caused by higher BNIP3/‐L levels could be reversed by silencing BNIP3. Thus, a better understanding of oral CSC biology and how BNIP3/‐L‐driven mitophagy contributes to the viability and stem‐like features of CSCs under hypoxic conditions is critical, suggesting a novel therapeutic strategy involving the inhibition of BNIP3 and autophagy in oral CSCs (Figure 10).

The pharmacological advances for cancer treatments depend on the commonsense hypothesis that increased cell proliferation and growth should be counterbalanced by a higher rate of apoptosis. Thus, apoptosis is one of the most common targeted pathways by current therapies. As confirmed in the literature, mitochondrial dynamics contribute differentially to diverse types of tumours, yet it is controversial whether mitochondrial fission or fusion supports cancer progression or apoptosis. To address this open question, first, we examined the cells' proliferation, cytotoxicity, and Caspase‐3 activity in our in vitro hypoxia model of oral cancer cells. We found that under hypoxic conditions, OECM‐1 CSCs are characterised by higher cell proliferation and EMT features with lower levels of caspase‐3 activity, suggesting that OECM‐1 CSCs exhibited a high potential to survive under a hypoxic stress environment compared to OECM‐1 cells.

As confirmed in the literature, mitochondrial dysfunction is a prerequisite for mitophagy by encouraging the engulfment of dysfunctional organelles through autophagosomes [37, 38], and it is considered an important feature of cancer [39, 40]. Accordingly, we found that in hypoxic conditions, resistant OECM‐1 CSCs are characterised by a fragmented mitochondrial phenotype, associated with higher levels of pro‐fission markers DRP1 and pDRP1 and insignificant changes in the pro‐fusion proteins OPA1 and MFN1. Interestingly, we show an opposing trend of another homologue of MFN1: MFN2, suggesting an additional, fusion‐independent role of MFN2 in ER tethering to the mitochondria. Proximity between the two organelles is implicated in several cellular processes, including mitochondrial fission and autophagosome formation. Consistent with an earlier study, wherein both mitochondrial fragmentation and the number of juxtaposition sites are increased in both CDDP‐resistant ovarian and osteosarcoma cancer cells, thereby enhancing the rates of mitochondrial fission and autophagosome biogenesis [29].

The development of a novel molecular‐targeted therapeutic approach provides new promise for advanced cancer treatments through the inhibition of early mitophagy/autophagy steps since mitophagy and autophagy are known to promote cancer progression and exhibit cytoprotective effects from different stress environments such as hypoxia. Cancer stem cells (CSCs) are currently acknowledged to demonstrate a high potential for cell survival under hypoxic stress conditions. The interplay between stress‐responsive mechanisms and autophagy in response to stress conditions is still unclear. Thus, elucidating the molecular mechanism by which increased mitophagy/autophagy systems can promote oral CSC malignancy, and their survival under stress conditions is essential. Interestingly, for the first time, we showed that BNIP3/‐L, a key adapter molecule in receptor‐mediated mitophagy machinery, has higher expression levels in the OECM‐1 CSCs than in OECM‐1 cells under hypoxic conditions, suggesting its potential role in the activation of autophagy‐associated signalling in CSCs. Different studies, including in vitro and in vivo, reported conflicting roles for mitophagy receptors and signalling regulators in cancer. In these studies, the authors showed that BNIP3 loss due to impaired receptor‐mediated mitophagy in murine breast cancer cells promoted malignancy resulting in higher accumulation of mitochondrial dysfunctions and increased oxidative stress [41, 42]. Nevertheless, several other types of cancers represented a strong positive correlation between their aggressive phenotype and higher BNIP3 levels, suggesting the immediate involvement of receptor‐mitophagy machinery in another mitophagy‐independent process, i.e., mitochondrial metabolism and cell motility and invasion properties, as in the case of BNIP3 [43, 44]. Consistent with these observations, we also analysed the preclinical database of UALCAN TCGA, wherein there is a positive correlation of higher BNIP3 expression with lower survival in four groups of HNSC patients. Further, in our study, we expand the role of BNIP3 in the context of oral CSC maintenance and survival, highlighting that oral CSCs exploit BNIP3‐driven mitophagy to survive under hypoxic stress environments by influencing mitochondrial OXPHOS metabolism, EMT, and acquisition of stem‐like functions. Consistently, we further demonstrated that the ablation of BNIP3 reduces the potential for oral CSC survival and stemness features under hypoxic conditions by blunting the mitophagy.

Recently, several studies also revealed that mitophagy plays an important role in energy homeostasis and is critical for CSC functions [30, 35]. The upregulated intracellular energy metabolic pathways in different CSCs for survival and growth have been studied intensively. However, it remains unclear how oral CSCs modulate their intracellular metabolism under hypoxic conditions. We performed a careful comparison of energy metabolism phenotypes between OECM‐1 and OECM‐1 CSCs, focusing on glycolysis and OXPHOS. Our study showed that BNIP3‐dependent mitophagy augmented OXPHOS metabolism reprogramming in OECM‐1 CSCs more than in OECM‐1 cells under hypoxic adverse conditions and that more OXPHOS, measured by OCR ETC protein activity, is converted into ATP through ATP synthase, indicating increased OXPHOS metabolism reprogramming in mitochondria. Over the past years, accumulating evidence on metabolism indicates that OXPHOS in some cancer stem cells is a critical process for survival and fitness, that higher OXPHOS activity in the microenvironment can significantly increase the numbers of CSCs in tumour tissue, and that functionally impaired OXPHOS activity leads to CSC demise in in vitro culture [45, 46, 47]. The major source of intracellular energy requirements for cancer cells is mitochondrial OXPHOS, which utilises the ETC to generate energy from glucose in ATP form. The reduction of ETC activity decreases the oxygen consumption rate, resulting in the inhibition of ATP synthesis. Numerous studies have shown that CSCs with high self‐renewal and metastatic potential are often accompanied by robust increases in OCR, ETC activity, and higher ATP levels, which induce CSCs to detach from the basement membrane in tumour tissues to form metastatic tumours [46]. Consistent with these previous studies, we also found that the higher activity of OCR (basal and maximum respiration capacity), ETC, and ATP levels in the OECM‐1 CSCs compared to OECM‐1 cells under hypoxic conditions indicates that increased OXPHOS metabolism endows oral CSCs with the ability to adapt in hypoxic environments, providing specific survival advantages. Indeed, higher OXPHOS metabolic activities support the conversion of differentiated cells into stem‐like cells and the maintenance of CSC‐ like phenotypes in pancreatic ductal adenocarcinoma (PDAC) stem cells [48], leukaemic stem cells (LSCs) [49], glioma stem cells (GSCs) [50] and hepatocellular carcinoma stem cells (HCSCs) [47]. Similarly, other studies have reported that increased oxidative metabolism is essential to sustain the biological properties of CSCs, specifically chemoresistance, self‐renewal, metastasis, and stemness‐like characteristics. It has also been reported in earlier studies that naïve and differentiating pluripotent stem cells primarily use OXPHOS to meet their energy requirements, while primed pluripotent stem cells favourably rely on glycolytic metabolism [51]. In line with this, we speculated that under hypoxia, oral CSCs, like naïve and differentiating pluripotent stem cells, use OXPHOS metabolism to fulfil their energy requirements and ensure their survival and stem cell fate. Furthermore, we elucidated the link between BNIP3‐dependent mitophagy and OXPHOS metabolism by silencing the BNIP3 expression. We observed that the ablation of BNIP3 levels in oral CSCs reverses the higher activity of OCR and, ETC, leading to a decrease in ATP levels due to the inhibition of OXPHOS metabolism, as this results in a reduction in the metastatic potential of CSCs without affecting their proliferation capacity under similar hypoxic environments. These interesting results suggest that BNIP3‐driven mitophagy‐mediated mitochondrial metabolism reprogramming may function as a switch for phenotyping, transitioning from a proliferation to a metastasis state in oral CSCs wherein it enables cancer cells to adapt or escape microenvironmental stress, resulting in sustained survival and stemness‐like features. However, since oral CSCs rely on OXPHOS metabolism, inhibiting this process could have unwanted adverse effects on metastatic potential and stemness‐like features. Therefore, we are convinced that targeting BNIP3‐driven mitophagy is a promising therapeutic approach for cancer treatment.

In this study, increased BNIP3‐dependent mitophagy in oral CSCs promoted OXPHOS metabolism reprogramming, resulting in higher cell motility and metastatic features, consistent with previous studies. To the best of our knowledge, studies on the relationship between OXPHOS metabolism and EMT in CSCs are limited, and only one published study showed that mitochondria OXPHOS metabolism reprogramming through FUNDC1, an effector mitophagy protein, enables cancer cells to adapt to microenvironment stress via heightened cell motility and metastatic propensity, leading to an escape from such an unfavourable environment [52]. In our study, we examined the EMT marker proteins in both OECM‐1 and OECM‐1 CSCs under hypoxic conditions. Our results showed that the EMT process was aggravated in hypoxia‐exposed OECM‐1 CSCs compared to OECM‐1 cells, as evidenced by decreased E‐CADHERIN and increased N‐CADHERIN, FIBRONECTIN, GSK3‐α/β, VIMENTIN, SNAIL, SLUG and TWIST. To the best of our knowledge, this is the first study that showed EMT is increased in oral CSCs due to higher BNIP3‐driven mitophagy with OXPHOS metabolism reprogramming. Further, these changes could be partially reversed by silencing BNIP3 expression. Additionally, we proposed that aberrant generation of ROS negatively affects cell motility, invasion, and metastatic growth of oral CSCs under hypoxic conditions. Similar to findings from earlier published studies, here non‐toxic ROS generation in hypoxia‐treated oral CSCs stimulates mitochondrial fission, resulting in more organelle trafficking and cell migration. However, more severe oxidative stress conditions in OECM‐1 cells and OECM‐1 CSCs with siBNIP3 impair cell migration and EMT properties. Together, these observations are also consistent with other findings that a non‐toxic level of ROS promotes EMT transition and enables greater metastatic propagation [53, 54, 55, 56], whereas an augmented ROS level, especially H_2_O_2_, suppresses mitochondrial fission, cell movement, and metastasis in in vitro and in vivo cancer models [57, 58, 59]. In another way, some earlier studies considered that smaller and fragmented mitochondria travel faster and farther in cancer cells, where the repositioning of mitochondria at the leading edges of migrating cells results in greater cell movement, invasion, and metastasis dissemination [60, 61].

## 5 Conclusion

Together, our observations provide novel insights into the importance of mitophagy systems in oral CSC maintenance and survival under stressful environments such as hypoxia. Importantly, we found that a higher level of BNIP3/‐L in hypoxic oral CSCs, primarily via phosphorylation at Ser616 of DRP1, is a crucial factor responsible for the increased proliferation and stemness features of oral CSCs. Inhibition of mitophagy arising from the silencing of BNIP3 expression led to a higher level of mitochondrial ROS generation, reduced cancer cell survival, and ultimately, the inhibition of tumour progression and metastasis. We also define a novel molecular mechanism by which BNIP3‐driven autophagy sustains oral CSC progression and metastasis under hypoxia through a mixed glycolytic/OXPHOS metabolism with a prevalent increase in OXPHOS levels, making them more reliant on mitochondrial oxidative metabolism for survival. Further targeting of this pathway could restrict the survival of CSCs, thereby developing new therapeutic strategies for cancer cells.

## Author Contributions

**Xin Li:** conceptualization (equal), data curation (equal), formal analysis (equal), methodology (equal), software (equal), validation (equal), writing – original draft (equal), writing – review and editing (equal). **Hitesh Singh Chaouhan:** conceptualization (equal), data curation (equal), formal analysis (equal), methodology (equal), software (equal), validation (equal), writing – original draft (equal), writing – review and editing (equal). **Shao‐Hua Yu:** data curation (equal), formal analysis (equal), investigation (equal), methodology (equal). **I‐Kuan Wang:** data curation (equal), formal analysis (equal), investigation (equal), methodology (equal). **Tung‐Min Yu:** data curation (equal), formal analysis (equal), investigation (equal), methodology (equal). **Ya‐Wen Chuang:** data curation (equal), formal analysis (equal), investigation (equal), methodology (equal). **Kuen‐Bao Chen:** formal analysis (equal), methodology (equal). **Feng‐Yen Lin:** formal analysis (equal), methodology (equal). **Michael Yuan‐Chien Chen:** formal analysis (equal), methodology (equal). **Che‐Hao Hsu:** formal analysis (equal), methodology (equal). **Kuo‐Ting Sun:** conceptualization (equal), formal analysis (equal), funding acquisition (equal), project administration (equal), resources (equal), supervision (equal), writing – review and editing (equal). **Chi‐Yuan Li:** conceptualization (equal), funding acquisition (equal), project administration (equal), resources (equal), supervision (equal), writing – review and editing (equal).

## Conflicts of Interest

The authors declare no conflicts of interest.

## Data Availability

The datasets are available from the corresponding author upon reasonable request.

## References

[1] H. R. Singhavi , S. Chakrabarti , A. Singh , et al., “Comparison of the Seventh and Eighth Editions American Joint Committee Cancer Classification System in Oral Cavity Squamous Cell Cancers,” International Journal of Cancer 146, no. 12 (2020): 3379–3384. 31583706 10.1002/ijc.32720

[2] K. N. Hema , T. Smitha , H. S. Sheethal , and S. A. Mirnalini , “Epigenetics in Oral Squamous Cell Carcinoma,” Journal of Oral and Maxillofacial Pathology 21, no. 2 (2017): 252–259. 28932035 10.4103/jomfp.JOMFP_150_17 PMC5596676

[3] A. T. Hawkins , P. E. Wise , T. Chan , et al., “Diverticulitis: An Update From the Age Old Paradigm,” Current Problems in Surgery 57, no. 10 (2020): 100862. 33077029 10.1016/j.cpsurg.2020.100862 PMC7575828

[4] S. Warnakulasuriya , “Global Epidemiology of Oral and Oropharyngeal Cancer,” Oral Oncology 45, no. 4–5 (2009): 309–316. 18804401 10.1016/j.oraloncology.2008.06.002

[5] V. Ernani and N. F. Saba , “Oral Cavity Cancer: Risk Factors, Pathology, and Management,” Oncology 89, no. 4 (2015): 187–195. 26088938 10.1159/000398801

[6] S. J. Davis , V. Divi , J. H. Owen , et al., “Metastatic Potential of Cancer Stem Cells in Head and Neck Squamous Cell Carcinoma,” Archives of Otolaryngology – Head & Neck Surgery 136, no. 12 (2010): 1260–1266. 21173377 10.1001/archoto.2010.219 PMC3315371

[7] X. Chu , W. Tian , J. Ning , et al., “Cancer Stem Cells: Advances in Knowledge and Implications for Cancer Therapy,” Signal Transduction and Targeted Therapy 9, no. 1 (2024): 170. 38965243 10.1038/s41392-024-01851-y PMC11224386

[8] M. L. O'Connor , D. Xiang , S. Shigdar , et al., “Cancer Stem Cells: A Contentious Hypothesis Now Moving Forward,” Cancer Letters 344, no. 2 (2014): 180–187. 24333726 10.1016/j.canlet.2013.11.012

[9] T. Wang , S. Shigdar , M. P. Gantier , et al., “Cancer Stem Cell Targeted Therapy: Progress Amid Controversies,” Oncotarget 6, no. 42 (2015): 44191–44206. 26496035 10.18632/oncotarget.6176 PMC4792551

[10] F. Y. Du , Q. F. Zhou , W. J. Sun , and G. L. Chen , “Targeting Cancer Stem Cells in Drug Discovery: Current State and Future Perspectives,” World Journal of Stem Cells 11, no. 7 (2019): 398–420. 31396368 10.4252/wjsc.v11.i7.398 PMC6682504

[11] R. T. Tavaluc , L. S. Hart , D. T. Dicker , and W. S. El‐Deiry , “Effects of Low Confluency, Serum Starvation and Hypoxia on the Side Population of Cancer Cell Lines,” Cell Cycle 6, no. 20 (2007): 2554–2562. 17912032 10.4161/cc.6.20.4911

[12] E. Lee , J. Yang , M. Ku , et al., “Metabolic Stress Induces a Wnt‐Dependent Cancer Stem Cell‐Like State Transition,” Cell Death & Disease 6, (7) (2015): e1805. 26136078 10.1038/cddis.2015.171 PMC4650724

[13] K. Palikaras and N. Tavernarakis , “Mitochondrial Homeostasis: The Interplay Between Mitophagy and Mitochondrial Biogenesis,” Experimental Gerontology 56 (2014): 182–188. 24486129 10.1016/j.exger.2014.01.021

[14] E. White , “The Role for Autophagy in Cancer,” Journal of Clinical Investigation 125, no. 1 (2015): 42–46. 25654549 10.1172/JCI73941 PMC4382247

[15] S. Campello and L. Scorrano , “Mitochondrial Shape Changes: Orchestrating Cell Pathophysiology,” EMBO Reports 11, no. 9 (2010): 678–684. 20725092 10.1038/embor.2010.115 PMC2933866

[16] C. M. Kenific and J. Debnath , “Cellular and Metabolic Functions for Autophagy in Cancer Cells,” Trends in Cell Biology 25, no. 1 (2015): 37–45. 25278333 10.1016/j.tcb.2014.09.001 PMC4275311

[17] T. J. Humpton , B. Alagesan , G. M. DeNicola , et al., “Oncogenic KRAS Induces NIX‐Mediated Mitophagy to Promote Pancreatic Cancer,” Cancer Discovery 9, no. 9 (2019): 1268–1287. 31263025 10.1158/2159-8290.CD-18-1409 PMC6726540

[18] Y. Xie , J. Liu , R. Kang , and D. Tang , “Mitophagy in Pancreatic Cancer,” Frontiers in Oncologia 11 (2021): 616079. 10.3389/fonc.2021.616079 PMC7953903 33718171

[19] P. P. Naik , S. Mukhopadhyay , P. K. Panda , et al., “Autophagy Regulates Cisplatin‐Induced Stemness and Chemoresistance via the Upregulation of CD44, ABCB1 and ADAM17 in Oral Squamous Cell Carcinoma,” Cell Proliferation 51 (2018): e12411. 29171106 10.1111/cpr.12411 PMC6528880

[20] C. Yan , L. Luo , C. Y. Guo , et al., “Doxorubicin‐Induced Mitophagy Contributes to Drug Resistance in Cancer Stem Cells From HCT8 Human Colorectal Cancer Cells,” Cancer Letters 388 (2017): 34–42. 27913197 10.1016/j.canlet.2016.11.018

[21] L. Hardy , M. Frison , and M. Campanella , “Breast Cancer Cells Exploit Mitophagy to Exert Therapy Resistance,” Oncotarget 9, no. 18 (2018): 14040–14041. 29581824 10.18632/oncotarget.24533 PMC5865650

[22] M. F. Mustafa , S. M. Saliluddin , S. Fakurazi , et al., “Expression of Autophagy and Mitophagy Markers in Breast Cancer Tissues,” Frontiers in Oncologia 11 (2021): 612009. 10.3389/fonc.2021.612009 PMC8416475 34490076

[23] C. Kurtman , M. Öztatlıcı , M. Üçöz , Ö. K. Çelik , I. Sokur , and M. K. Özbilgin , “Mitophagy in the A549 Lung Cancer Cell Line, Radiation‐Induced Damage, and the Effect of ATM and PARKIN on the Mitochondria,” International Journal of Radiation Research 20, no. 1 (2022): 9–13.

[24] K. Liu , J. Lee , J. Y. Kim , et al., “Mitophagy Controls the Activities of Tumor Suppressor p53 to Regulate Hepatic Cancer Stem Cells,” Molecular Cell 68, no. 2 (2017): 281–292.e5. 29033320 10.1016/j.molcel.2017.09.022 PMC5687282

[25] X. Chen , J. Gong , H. Zeng , et al., “MicroRNA145 Targets BNIP3 and Suppresses Prostate Cancer Progression,” Cancer Research 70, no. 7 (2010): 2728–2738. 20332243 10.1158/0008-5472.CAN-09-3718

[26] M. I. Koukourakis , A. Giatromanolaki , A. Polychronidis , et al., “Endogenous Markers of Hypoxia/Anaerobic Metabolism and Anemia in Primary Colorectal Cancer,” Cancer Science 97, no. 7 (2006): 582–588. 16827797 10.1111/j.1349-7006.2006.00220.x PMC11159659

[27] E. Y. Tan , L. Campo , C. Han , et al., “BNIP3 as a Progression Marker in Primary Human Breast Cancer; Opposing Functions in In Situ Versus Invasive Cancer,” Clinical Cancer Research 13, no. 2 Pt 1 (2007): 467–474. 17255267 10.1158/1078-0432.CCR-06-1466

[28] A. Giatromanolaki , M. I. Koukourakis , K. C. Gatter , A. L. Harris , and E. Sivridis , “BNIP3 Expression in Endometrial Cancer Relates to Active Hypoxia Inducible Factor 1alpha Pathway and Prognosis,” Journal of Clinical Pathology 61, no. 2 (2008): 217–220. 17513511 10.1136/jcp.2007.046680

[29] C. Vianello , V. Cocetta , D. Catanzaro , et al., “Cisplatin Resistance Can Be Curtailed by Blunting BNIP3‐Mediated Mitochondrial Autophagy,” Cell Death & Disease 13, (4) (2022): 398. 35459212 10.1038/s41419-022-04741-9 PMC9033831

[30] K. M. Dykstra , H. R. S. Fay , A. C. Massey , et al., “Inhibiting Autophagy Targets Human Leukemic Stem Cells and Hypoxic AML Blasts by Disrupting Mitochondrial Homeostasis,” Blood Advances 5, no. 8 (2021): 2087–2100. 33877295 10.1182/bloodadvances.2020002666 PMC8095145

[31] S. H. Yu , K. Palanisamy , K. T. Sun , et al., “Human Antigen R Regulates Hypoxia‐Induced Mitophagy in Renal Tubular Cells Through PARKIN/BNIP3L Expressions,” Journal of Cellular and Molecular Medicine 25, no. 5 (2021): 2691–2702. 33496385 10.1111/jcmm.16301 PMC7933924

[32] D. Liang , Y. Ma , J. Liu , et al., “The Hypoxic Microenvironment Upgrades Stem‐Like Properties of Ovarian Cancer Cells,” BMC Cancer 12 (2012): 201. 22642602 10.1186/1471-2407-12-201 PMC3407800

[33] H. S. Chaouhan , X. Li , K. T. Sun , et al., “Calycosin Alleviates Paraquat‐Induced Neurodegeneration by Improving Mitochondrial Functions and Regulating Autophagy in a *Drosophila* Model of Parkinson's Disease,” Antioxidants (Basel) 11, no. 2 (2022): 222. 35204105 10.3390/antiox11020222 PMC8868496

[34] V. Madhu , M. Hernandez‐Meadows , P. K. Boneski , et al., “The Mitophagy Receptor BNIP3 is Critical for the Regulation of Metabolic Homeostasis and Mitochondrial Function in the Nucleus Pulposus Cells of the Intervertebral Disc,” Autophagy 19, no. 6 (2023): 1821–1843. 36628478 10.1080/15548627.2022.2162245 PMC10262801

[35] Y. Y. Chen , W. H. Wang , L. Che , et al., “BNIP3L‐Dependent Mitophagy Promotes HBx‐Induced Cancer Stemness of Hepatocellular Carcinoma Cells via Glycolysis Metabolism Reprogramming,” Cancers (Basel) 12 (2020): 3. 10.3390/cancers12030655 PMC7139741 32168902

[36] D. S. Chandrashekar , B. Bashel , S. A. H. Balasubramanya , et al., “UALCAN: A Portal for Facilitating Tumor Subgroup Gene Expression and Survival Analyses,” Neoplasia 19, no. 8 (2017): 649–658. 28732212 10.1016/j.neo.2017.05.002 PMC5516091

[37] S. P. Elmore , T. Qian , S. F. Grissom , and J. J. Lemasters , “The Mitochondrial Permeability Transition Initiates Autophagy in Rat Hepatocytes,” FASEB Journal 15, no. 12 (2001): 2286–2287. 11511528 10.1096/fj.01-0206fje

[38] L. C. Gomes and L. Scorrano , “Mitochondrial Morphology in Mitophagy and Macroautophagy,” Biochimica et Biophysica Acta 1833, no. 1 (2013): 205–212. 22406072 10.1016/j.bbamcr.2012.02.012

[39] S. Grandemange , S. Herzig , and J.‐C. Martinou , “Mitochondrial Dynamics and Cancer,” Seminars in Cancer Biology 2009 (2009): 50–56. 10.1016/j.semcancer.2008.12.001 19138741

[40] J. Guo , F. Ye , X. Jiang , et al., “Drp1 Mediates High Glucose‐Induced Mitochondrial Dysfunction and Epithelial‐Mesenchymal Transition in Endometrial Cancer Cells,” Experimental Cell Research 389 (2020): 111880. 32017930 10.1016/j.yexcr.2020.111880

[41] A. H. Chourasia , K. Tracy , C. Frankenberger , et al., “Mitophagy Defects Arising From BNIP3 Loss Promote Mammary Tumor Progression to Metastasis,” EMBO Reports 16, no. 9 (2015): 1145–1163. 26232272 10.15252/embr.201540759 PMC4576983

[42] A. Lyons , M. Coleman , S. Riis , et al., “Insulin‐Like Growth Factor 1 Signaling Is Essential for Mitochondrial Biogenesis and Mitophagy in Cancer Cells,” Journal of Biological Chemistry 292, no. 41 (2017): 16983–16998. 28821609 10.1074/jbc.M117.792838 PMC5641874

[43] S. Maertin , J. M. Elperin , E. Lotshaw , et al., “Roles of Autophagy and Metabolism in Pancreatic Cancer Cell Adaptation to Environmental Challenges,” American Journal of Physiology. Gastrointestinal and Liver Physiology 313, no. 5 (2017): G524–G536. 28705806 10.1152/ajpgi.00138.2017 PMC5792215

[44] M. Vara‐Perez , H. Maes , S. Van Dingenen , and P. Agostinis , “BNIP3 Contributes to the Glutamine‐Driven Aggressive Behavior of Melanoma Cells,” Biological Chemistry 400, no. 2 (2019): 187–193. 29924728 10.1515/hsz-2018-0208

[45] G. Farnie , F. Sotgia , and M. P. Lisanti , “High Mitochondrial Mass Identifies a Sub‐Population of Stem‐Like Cancer Cells That Are Chemo‐Resistant,” Oncotarget 6, no. 31 (2015): 30472–30486. 26421710 10.18632/oncotarget.5401 PMC4741545

[46] V. S. LeBleu , J. T. O'Connell , K. N. Gonzalez Herrera , et al., “PGC‐1α Mediates Mitochondrial Biogenesis and Oxidative Phosphorylation in Cancer Cells to Promote Metastasis,” Nature Cell Biology 16, no. 10 (2014): 992–1003. 25241037 10.1038/ncb3039 PMC4369153

[47] G. Liu , Q. Luo , H. Li , Q. Liu , Y. Ju , and G. Song , “Increased Oxidative Phosphorylation Is Required for Stemness Maintenance in Liver Cancer Stem Cells From Hepatocellular Carcinoma Cell Line HCCLM3 Cells,” International Journal of Molecular Sciences 21, no. 15 (2020): 5276. 32722385 10.3390/ijms21155276 PMC7432880

[48] S. Valle , S. Alcalá , L. Martin‐Hijano , et al., “Exploiting Oxidative Phosphorylation to Promote the Stem and Immunoevasive Properties of Pancreatic Cancer Stem Cells,” Nature Communications 11, no. 1 (2020): 5265. 10.1038/s41467-020-18954-z PMC7567808 33067432

[49] I. Baccelli , Y. Gareau , B. Lehnertz , et al., “Mubritinib Targets the Electron Transport Chain Complex I and Reveals the Landscape of OXPHOS Dependency in Acute Myeloid Leukemia,” Cancer Cell 36, no. 1 (2019): 84–99.e8. 31287994 10.1016/j.ccell.2019.06.003

[50] E. Vlashi , C. Lagadec , L. Vergnes , et al., “Metabolic State of Glioma Stem Cells and Nontumorigenic Cells,” Proceedings of the National Academy of Sciences of the United States of America 108, no. 38 (2011): 16062–16067. 21900605 10.1073/pnas.1106704108 PMC3179043

[51] N. Shyh‐Chang and H. H. Ng , “The Metabolic Programming of Stem Cells,” Genes & Development 31, no. 4 (2017): 336–346. 28314766 10.1101/gad.293167.116 PMC5358754

[52] J. Li , E. Agarwal , I. Bertolini , et al., “The Mitophagy Effector FUNDC1 Controls Mitochondrial Reprogramming and Cellular Plasticity in Cancer Cells,” Science Signaling 13 (2020): 642. 10.1126/scisignal.aaz8240 PMC7484983 32723812

[53] A. Bezawork‐Geleta , E. J. Brodie , D. A. Dougan , and K. N. Truscott , “LON Is the Master Protease That Protects Against Protein Aggregation in Human Mitochondria Through Direct Degradation of Misfolded Proteins,” Scientific Reports 5 (2015): 17397. 26627475 10.1038/srep17397 PMC4667172

[54] M. C. Caino , J. H. Seo , A. Aguinaldo , et al., “A Neuronal Network of Mitochondrial Dynamics Regulates Metastasis,” Nature Communications 7 (2016): 13730. 10.1038/ncomms13730 PMC5187409 27991488

[55] J. C. Ghosh , J. H. Seo , E. Agarwal , et al., “Akt Phosphorylation of Mitochondrial Lonp1 Protease Enables Oxidative Metabolism and Advanced Tumor Traits,” Oncogene 38, no. 43 (2019): 6926–6939. 31406245 10.1038/s41388-019-0939-7 PMC6814529

[56] T. R. Hurd , M. DeGennaro , and R. Lehmann , “Redox Regulation of Cell Migration and Adhesion,” Trends in Cell Biology 22, no. 2 (2012): 107–115. 22209517 10.1016/j.tcb.2011.11.002 PMC4515034

[57] V. Debattisti , A. A. Gerencser , M. Saotome , S. Das , and G. Hajnóczky , “ROS Control Mitochondrial Motility Through p38 and the Motor Adaptor Miro/Trak,” Cell Reports 21, no. 6 (2017): 1667–1680. 29117569 10.1016/j.celrep.2017.10.060 PMC5710826

[58] K. Ishikawa , K. Takenaga , M. Akimoto , et al., “ROS‐Generating Mitochondrial DNA Mutations Can Regulate Tumor Cell Metastasis,” Science 320, no. 5876 (2008): 661–664. 18388260 10.1126/science.1156906

[59] E. Piskounova , M. Agathocleous , M. M. Murphy , et al., “Oxidative Stress Inhibits Distant Metastasis by Human Melanoma Cells,” Nature 527, no. 7577 (2015): 186–191. 26466563 10.1038/nature15726 PMC4644103

[60] B. Cunniff , A. J. McKenzie , N. H. Heintz , and A. K. Howe , “AMPK Activity Regulates Trafficking of Mitochondria to the Leading Edge During Cell Migration and Matrix Invasion,” Molecular Biology of the Cell 27, no. 17 (2016): 2662–2674. 27385336 10.1091/mbc.E16-05-0286 PMC5007087

[61] J. Zhao , J. Zhang , M. Yu , et al., “Mitochondrial Dynamics Regulates Migration and Invasion of Breast Cancer Cells,” Oncogene 32, no. 40 (2013): 4814–4824. 23128392 10.1038/onc.2012.494 PMC3911914

